# Social and Cultural Constraints on Football Player Development in Stockholm: Influencing Skill, Learning, and Wellbeing

**DOI:** 10.3389/fspor.2022.832111

**Published:** 2022-05-20

**Authors:** James Vaughan, Clifford J. Mallett, Paul Potrac, Carl Woods, Mark O'Sullivan, Keith Davids

**Affiliations:** ^1^The University of Queensland, School of Human Movement and Nutrition Sciences, Brisbane, QLD, Australia; ^2^Allmänna Idrottsklubben (AIK) FC Stockholm, Research and Development Department, Stockholm, Sweden; ^3^Technical University of Munich, Faculty of Sport and Health Sciences, München, Germany; ^4^Northumbria University, Department of Sport, Exercise and Rehabilitation, Newcastle upon Tyne, United Kingdom; ^5^Institute for Health and Sport, Victoria University, Melbourne, VIC, Australia; ^6^Sport and Human Performance Research Group, Sheffield Hallam University, Sheffield, United Kingdom

**Keywords:** ecological dynamics, skilled intentionality, ethnography, transdisciplinarity, talent development, Athlete Talent Development Environment, ecological values, sport coaching

## Abstract

In this paper, we consider how youth sport and (talent) development environments have adapted to, and are constrained by, social and cultural forces. Empirical evidence from an 18-month ethnographic case study highlights how social and cultural constraints influence the skill development and psychological wellbeing of young football players. We utilized novel ways of knowing (i.e., epistemologies) coupled to ecological frameworks (e.g., the theory of ecological dynamics and the skilled intentionality framework). A transdisciplinary inquiry was used to demonstrate that the values which athletes embody in sports are *constrained* by the *character* of the social institutions (sport club, governing body) and the social order (culture) in which they live. The *constraining character* of an athlete (talent) development environment is captured using ethnographic methods that illuminate a sociocultural value-directedness toward *individual competition*. The discussion highlights how an emphasis on *individual competition* overshadows opportunities (e.g., shared, and nested affordances) for *collective collaboration* in football. Conceptually, we argue that these findings characterize how a dominating sociocultural constraint may negatively influence the skill development, in game performance, and psychological wellbeing (*via* performance anxiety) of young football players in Stockholm. Viewing cultures and performance environments as embedded complex adaptive systems, with human development as ecological, it becomes clear that microenvironments and embedded relations underpinning athlete development in high performance sports organizations are deeply susceptible to broad cultural trends toward neoliberalism and competitive individualism. Weaving transdisciplinary lines of inquiry, it is clarified how a value directedness toward *individual competition* may overshadow *collective collaboration*, not only amplifying socio-cognitive related issues (anxiety, depression, emotional disturbances) but simultaneously limiting perceptual learning, skill development, team coordination and performance at all levels in a sport organization.

## Introduction

Football may begin with just having fun, but it can become far more serious stuff, as any English hooligan or Argentinian nationalist will attest. Football can formulate personal identities, it can cement large scale communities, and it can even provide reasons for violence. Nations and religions are football clubs on steroids (Harari, 2018, p. 241).

In this paper, we examine how the social and cultural *seriousness* associated with football acts as a constraint on player development, cascading into athlete development environments, and influencing players' psychological wellbeing and skill development. Gaining detailed insights from a high performance sports organization, we consider how youth sport has adapted to, and is constrained by, social and cultural forces, highlighting how “task, environment, and performer constraints are fundamentally influenced by sociocultural constraints” (Vaughan et al., 2019, p. 7; see also Araújo et al., 2010; Araújo and Davids, 2011). Aligned to the framework of Ecological Dynamics, we view athlete development and team performance as inherently complex and continuously adaptive to an ensemble of changing constraints (Chow et al., 2007; Davids, 2015; Button et al., 2020). In concert with these perspectives, we illustrate how the values that players embody, and the skills they develop, are coupled to, and *constrained* by, the *character* of the social institutions (sport club, governing body) and the social order (culture) in which they live (Schwartz, 1990; Vaughan et al., 2021).

To investigate the interacting constraints that continually shape relations between a society, culture, community and peoples psychological wellbeing, and skill development, we combine ecological approaches with transdisciplinary inquiry (Vaughan et al., 2019; Woods et al., 2021a). Transcending disciplinary boundaries allows us to function in-between, through and beyond disciplinary conventions (Mahan, 1970; Woods et al., 2021b), placing the phenomenon of interest, in this case, football player development, at the core of the research program, not a disciplinary way of doing or being *per se* (Montuori, 2013). Transdisciplinarity allows us to extend ecological approaches and illuminate the *intentionality of a player-environment ecology*, rather than merely highlighting the intentions of individual athletes (Vaughan et al., 2021). In particular, we aim to examine the extent to which skilled responsiveness to *affordances* (opportunities for action; Gibson, 1979) on the football pitch is value-directed and value realizing, arising within the intentionality developed in a specific environment (Hodges and Baron, 1992; Vaughan et al., 2021).

To achieve this aim, an 18-month ethnographic case study into youth football in Stockholm was undertaken. Informed by an Ecological Dynamics rationale and the conceptual Skilled Intentionality Framework (van Dijk and Rietveld, 2017; Button et al., 2020), the study also employed the Athlete Talent Development Environment (ATDE: Henriksen et al., 2010) as a framework for data collection and organization. Data were gathered, analyzed, and synthesized, using these frameworks to examine the notion that value-directedness of player-environment intentionality acts as a sociocultural constraint on skill development. More directly, we explored the extent to which the value-directedness that players experience on the football pitch is related to the macrosystems, sociocultural constraints, and forms of life that influence responsiveness to affordances and psychological wellbeing (Rietveld and Kiverstein, 2014). This case study highlights the extent to which a specific player development ecology, or ATDE is influenced by social and cultural constraints, and how such constraints shape the development (learning, wellbeing, and performance) of young football players. The approach taken is refined, and expanded in the Learning in Development Research Framework (see O'Sullivan et al., 2021).

## A Contextual Analysis

First, we provide some background on the need for a transdisciplinary and ecological perspective by highlighting the extent to which social, cultural, political, historical, and scientific forces have led most sport science and talent development research to marginalize athlete-environment relations in favor of an inordinate focus on the individual only (evidenced as the *organismic asymmetry* bias, see Davids and Araújo, 2010). To outline this argument, we present a contextual analysis of the ecological context called the *macrosystem* (Bronfenbrenner, 2005; Uehara et al., 2016). As the overarching context in Bronfenbrenner's bioecological theory of human development, the macrosystem shapes a societal blueprint, conveying the information, ideology, and values that influence organizational structures (i.e., roles, responsibilities, tasks) and events in the embedded microsystems (i.e., classrooms and coaching sessions) where childhood development and education takes place (Kasser and Linn, 2016). Indeed, to better understand athlete development in and through sport, culture and context matter most (Araújo et al., 2017; Juarrero, 2019; O'Sullivan et al., 2021; Vaughan et al., 2021).

### The Culture of Youth Sport

There have been many examples of instances of physical and psychological harm, as well as a culture of bullying, which have troubled youth sport in many different societies (e.g., Palmgren, 2018; Nery et al., 2019, 2020; Aguilar et al., 2020). Recent investigations into professional football academies in the United Kingdom highlighted these harmful influences, with hundreds of young players reporting abuse and ongoing mental health problems^1^ (BBC, 2021; Scott, 2021). While mental health problems are not isolated to sport (Sebbens et al., 2016), a question of concern is how the culture of youth sport amplifies broader issues, with sport being a microcosm of a society in which it is embedded. In Canada, US, and the UK, Curran and Hill (2019) identified three broad cultural changes that are adversely affecting the psychological wellbeing and mental health of young people born in the last 35 years: (i) the emergence of neoliberalism and competitive individualism, (ii) the doctrine of meritocracy, and (iii), increasingly anxious and controlling parental practices (Curran and Hill, 2019). Co-adapting to these cultural changes, youth sport has become increasingly adult-oriented, professionalized and media-centered, leading many to argue that children are being treated as “mini-adults” and molded to conform to “professionalized standards” (Bergeron et al., 2015; Fahlström et al., 2015).

### A Neoliberal Corporate Capitalist Macrosystem

Margaret thatcher once said that:

…it isn't that I set out on economic policies; it's that I set out really to change the approach, and changing the economics is the means of changing that approach. If you change the approach you really are after the heart and soul of the nation. Economics are the method; the object is to change the heart and soul.” (The Sunday Times, 1981)

The globalization of neoliberal policies and the spread of American Corporate Capitalism (ACC: see Kasser et al., 2007 for detailed distinctions) have led scholars to argue that a neoliberal corporate capitalist macrosystem is adversely affecting childhood development environments (Kasser and Linn, 2016; McKay and Miller, 2016). In particular, it is argued that neoliberalism has succeeded in recalibrating cultural values to over-emphasize competitiveness and individualism above all else (Curran and Hill, 2019). This political shift in ideology and values is evident in classrooms and educational institutions, as increasing pressures to perform, conform, comply and compete prompt schools and teachers to adopt controlling managerial practices that stifle learning and creativity (Taylor et al., 2008; Halffman and Radder, 2015; Vaughan et al., 2019). Compounding these issues, the materialistic value orientations of neoliberal capitalism amplify interpersonal competition and dampen the intention (and opportunity) to collaborate (Sheldon et al., 2000; Kasser et al., 2007). In certain contexts, advertising, social media profiles and profit-driven educational institutions (i.e., privatized schools and academies) intensify individual competition, hierarchy, and extrinsic rewards, often to the detriment of autonomous motivation and psychological wellbeing (Kasser and Linn, 2016). However, the ways in which the corporate capitalist macrosystem affects organizational structures and microenvironments differs between countries, cultures and contexts (Vaughan et al., 2019). This recognition highlights the need to carefully examine and document how macrosystem influences interact with embedded cultures and specific contexts to shape the microenvironments of athlete development (O'Sullivan et al., 2021).

### The Embedded Microenvironments of Athletic Development

Many athlete development (micro) environments are embedded within and influenced by the neoliberal corporate capitalist macrosystem (Henriksen and Stambulova, 2017; Vaughan et al., 2019). At all levels, sport performance environments are becoming increasingly entangled with the agendas of the media, advertising, politicians and multinational corporations (Bergeron et al., 2015). This messy entanglement has exposed young athletes to rampant forms of commodification promoting individual competition above all else (McKay and Miller, 2016). Evidence shows that the prioritization of monetary profit and power (i.e., the desire to dominate people and exploit resources) can suppress values that support the nurturing of children (Kasser and Linn, 2016). To the detriment of players and coaches in sport, neoliberalism has seen a traditional emphasis on collectivism (e.g. camaraderie, teamwork, an ethos of discovery and exploration) progressively give way to a wave of competitive individualism and corporate managerialism (Potrac et al., 2013, 2017; Curran and Hill, 2019; Gale et al., 2019; Ives et al., 2019; Vaughan, 2020). The prevalence of performance analytics and *Big Data* methodologies can amplify the issues, with statistics and metrics enhancing surveillance, intrusive rankings, and comparisons of performance to the point whereby teammates and colleagues are oft-seen as competitors (Kalfa et al., 2018). An obsessive focus on numbers—manifest, for example, in the number of social media followers or “likes” and/or performance objectives (i.e., meters run, number of passes, dribbles, tackles made etc.)—a form of “dataveillance,” becomes dehumanizing, over-individualizing team sports, affecting how athletes relate to each other and how they see themselves (Manley and Williams, 2019).

It is within these cultural contexts that the standard model of talent development (SMTD) has emerged. The SMTD is characterized by early selection into exclusive training programs that often promote hyper-specialization and result in eventual deselection (Fahlström et al., 2015; Bailey and Collins, 2016). While there are increasing calls for diverse sporting experiences beyond the SMTD (Cot and Ericsson, 2015; Santos et al., 2016), the majority of football academies remain anchored to this approach (Ford et al., 2012). The SMTD often exposes children to being evaluated, assessed, and categorized as “footballers” in infancy, and as “elite” under the age of five years (Austin, 2019), by adults perpetuating a Darwinian form of competition—i.e. survival of the fittest (e.g., see https://www.thenationalnews.com/world/uk-news/2021/10/21/four-year-old-footballer-scouted-by-arsenal-while-still-in-nursery/). Pressure to specialize, comply, perform, and conform, from a very early age, increases the risk of physical and psychological harm, often accelerating athletes toward events that lead to deselection (Bergeron et al., 2015; Fahlström et al., 2015; Ivarsson et al., 2018). According to the English football association, 99.5% of children selected into a Premier League academy (at under 9) are deselected (thefa, 2016). Studying 29 of the best professional football academies from around the world (Ford et al., 2020) exposed a high annual turnover, with around 29%, of players deselected through age groups every year. Further, deselection from academies has been associated with emotional and psychological disturbances, like anxiety, fear, depression, anger and humiliation (Brown and Potrac, 2009).

Henriksen et al. (2010) argued that modern societies have serious problems with the recruitment and transitions of athletes into professional sport (see Swainston et al., 2020, 2021); however, little has changed to address these issues. The moral bankruptcy of youth sport programmes seems to be deeply entwined with cultural and systemic transitions toward neoliberal ideology, rampant commodification, and extreme competitive individualism. While there is no doubt that these cultural influences have shaped athlete development practices and pathways away from notions of “play,” they have also reinforced an ontological naivety of sport; a naivety that has been aided and abetted by the continued creation and application of sport science research that is de-contextualized and de-humanized, the mono-disciplinarity of which narrows our ways of knowing by limiting our understanding of human behavior and development (Jones et al., 2010; Uehara et al., 2016; Balagué et al., 2017; Nelson, 2018; Vaughan et al., 2019, 2021; López-Felip et al., 2020; Vaughan, 2020). This disciplinary blinkering has led a majority of sport science and talent development practices (e.g., SMTD) to ignore the reality that, as in all human behaviors, fluctuations in development trajectories of athletes (and associated inclines, pauses and declines in performance) are naturally occurring aspects of a non-linear and complex development process. This common aspect of human development also characterizes the journey in sport (Balagué et al., 2017; Adolph, 2019; Button et al., 2020; Chow et al., 2022).

To overcome the decontextualising dominance of such research in sport, scholars are arguing for greater emphasis on transdisciplinary inquiry (Vaughan et al., 2019; Woods, 2021). In advocating for transdisciplinarity, we adopt a unified perspective to examine the extent to which the practices and methods within the SMTD increase the risks of *socio-cognitive related issues* (anxiety, depression, emotional disturbances) and are simultaneously limiting *perceptual learning, skill development* and *team coordination* in sport (Rietveld and Kiverstein, 2014; Woods et al., 2020; Vaughan et al., 2021). To achieve this aim, we introduce novel and alternative epistemologies that are coupled to, and may inform, an ecological view of athlete development. Ecological perspectives facilitate a radically alternative epistemology, based on the environment-person system being conceived as indeterminate, holistic and deeply integrated (Davids et al., 2012; Renshaw and Chow, 2019). Pedagogical practice, along with many other areas of professional expertise, has been shaped by the traditionally dominant scientific philosophy of reductionism, based on a deterministic (completely predictable) view of the world, dating back to the Enlightenment period in European history (Glimcher, 2005). Scientific determinism arose in the mid-seventeenth century because of the desire to reduce uncertainty and enhance predictive capacity in human societies and institutions. Ecological perspectives, in contrast, encourage an epistemology which advocates an indeterminate (not completely predictable over a longer timescale) worldview, in which James Gibson's (1979) *knowledge of* the environment is continuously emerging and paramount in guiding human interactions (Davids et al., 2012). Recognizing the apparent indeterminate nature of solutions to functional behaviors during sport performance and learning has amplified the relevance of relativist theoretical perspectives in more recent times (Glimcher, 2005). As we exemplify next, in adopting an ecological epistemology—context shapes everything…continually (Juarrero, 2019).

## An ATDE Needs to Be Sociomaterial and Coupled to Affordances of a Form of Life

ATDE researchers seek to shift the investigatory focus away from “the *individual* athletes,” toward “the broader development context or *environment* in which they develop” (Henriksen and Stambulova, 2017, p. 271). Utilizing the Skilled Intentionality Framework (van Dijk and Rietveld, 2017; SIF: see Rietveld et al., 2018) we extend this view, toward understanding behavior, learning and skill development at the ecological level of interactions between a performer and their performance environment, both continuously shaping each other (Button et al., 2020). The SIF provides philosophical foundations for pursuing such a shift. It describes the ontology of *constitutive sociomaterial entanglement*, that sees the ways in which we live (i.e., forms of life—described below), the practices we participate in (i.e., football), the affordances we perceive (i.e., Gibson, 1979; opportunities for action), and the skills we develop (i.e., passing, dribbling) as constitutive relations and aspects of the same whole that continuously (re)form each other (van Dijk and Rietveld, 2017; Vaughan et al., 2021). We propose that the ATDE can provide a perspective on the extent to which athlete development environments are sociomaterial and constitutively entangled (deeply nested and embedded) within broader (sociocultural and historical) macro contexts and structures (Henriksen et al., 2010; Henriksen and Stambulova, 2017). Understanding these ideas will help us focus on how context changes everything (Juarrero, 2019).

The SIF makes a profound contribution to the way one views an ATDE by detailing the extent to which the intentionality of any human-environment system (i.e., ATED/ecology) frames an interdependent and constitutive relationship (van Dijk and Rietveld, 2017). In other words:

Intentionality characterizes the system, not just biological organisms within the system. Thus, intentionality in the sense of value-directedness characterizes environmental structures [i.e., organizational structures within an ATDE] and processes [i.e., training sessions] as much as it does the organisms [football players] who shape and are shaped [e.g., skill development] by those structures and processes. This implies that values are necessary constraints on both the constitution and the selection of affordances (Hodges and Baron, 1992, pp. 269–270).

Hodges and Baron (1992) suggest that *value-directedness* provides insight into “the intentions of the world as a self-organizing system” (p. 270). This key idea draws attention to the important implication that, although self-organization processes can emerge spontaneously (Kelso, 1995), in human behavior, they are continually shaped by the specific constraints of each individual, immediate task and the social and physical environment (Button et al., 2020). In this way, value-directedness characterizes the intentionality of broad ecosystems and influences embedded athlete-environment systems. In the SIF, the intentionality of broader ecosystems is refined by the concept of a *form of life* (Wittgenstein, 1953)—described as regular behavioral patterns that arise in interaction with myriad constraints that transcend disciplinary boundaries (van Dijk and Rietveld, 2017; Vaughan et al., 2019). Reed (1993) called these regular patterns of behavior *embodied intentions*. He argued that *intentions* are not enclosed within an individual's body and insulated from the environment, but rather form a dynamic relation with an existing ecological niche. Extending these ideas, the SIF sees embodied intentions as the persistent ways in which people engage with relevant fields of affordances within a form of life, whereby skilled intentionality is defined as “the selective engagement with multiple affordances simultaneously in a concrete situation” (Rietveld et al., 2018, p. 1). Being shaped by myriad constraints, *forms of life* may be powerful sociocultural influences on normative behaviors (i.e., engagement with affordances) and customs of our communities, organizations and cultures (Vaughan et al., 2019). Such patterns of behavior emerge in desires, values, ideas and attitudes shown by individuals inhabiting a form of life (Rothwell et al., 2019).

Selectively engaging with multiple affordances simultaneously, is central to an ecological perspective on perceptual learning, skill development, team coordination, and the potential for creativity in team sports (Vaughan et al., 2019; Button et al., 2020; Chow et al., 2020). Emerging from “decades of research on affordances in the tradition of Ecological Psychology” (Rietveld et al., 2018, p. 5) the SIF helps illuminate how our environments are constitutively entangled in the relevant fields of affordances that *stand out* within a particular form of life (van Dijk and Rietveld, 2017; Rietveld et al., 2018). Indeed, “the constitution and detection of affordances is a partial realization of values” (Hodges and Baron, 1992, p. 263): whereby relevant fields of affordances invite the partial realization of social and cultural value in a context-sensitive and embodied way (Vaughan et al., 2021).

### Player-Environment Intentionality, Value-Directedness, and Affordances in Football

In football, a value-directedness (of intentionality) directs players' attention toward certain environmental properties (e.g., teammates, opponents, pitch markings, and goals, informing gaps and spaces), and therefore influences who or what is targeted, and which affordances are solicited, discovered, exploited, and/or invented (Rasmussen et al., 2017; Vaughan et al., 2021). Affordances and their related effectivities (Gibson, 1979: individual, capacities, capabilities/skills sets) can only be discovered and used when intentionality and lawfully-specified possibilities are coordinated (Turvey, 1990). Here, we contend that the relational properties of effectivities and affordances arise with the player-environment intentionality experienced by players, coupled to specific contexts within the game. Effectivities describe the properties of individual players which allows them to utilize a feature of the environment (e.g., “scanning” to exploit space or dribbling the ball). Affordances, on the other hand, describe properties of the environment to which a player pays attention and utilizes (e.g., an opportunity or an invitation through movement of a teammate to pass the ball forward) (Vaughan et al., 2021). More formally:

Situation X affords activity Y for organism Z on occasion O if, and only if, X and Z are mutually compatible on dimensions of relevance to Y.

Organism Z effects activity Y in situation X on occasion O if, and only if, Z and X are mutually compatible on dimensions of relevance to Y (Turvey et al., 1981; López-Felip and Turvey, 2017).

As an aspect of player-environment intentionality, in a sports organization, value-directedness plays a central role in educating attention of individual players toward environmental properties and shaping the relevant fields of affordances that are soliciting, and “standing out,” to be more readily perceived and acted upon (see Vaughan et al., 2021 for details). A particular issue to be clarified concerns how value-directedness shapes skill development opportunities, shining a light on certain affordances that “stand out” and simultaneously overshadowing other (potentially nested) affordances that remain imperceptible to learners in an organization [see (Vaughan et al., 2021) and (O'Sullivan et al., 2021) for a specific examples in football].

### Illustrating Value-Directedness: A Transdisciplinary Approach

Contrary to the perspective of socio-cognitive psychology put forth by Schwartz (1994) and Maio et al. (2009), we do not conceive values as *a priori* agreed entities to be imprinted upon, and represented within, the information processing mind. Instead, we adopt ecological perspectives on values (Hodges and Raczaszek-Leonardi, 2021), and view value-directedness as an adaptive, emergent and relational aspect of person-environment intentionality (Hodges and Baron, 1992; Vaughan et al., 2021). While socio-cognitive accounts have pre-supposed that values research might lead to *a priori* predictions or casual insinuations, our ecological ontology (emphasizing constitutive sociomaterial entanglement) is aligned to the sciences of complex adaptive systems. In this ecological account, emergent phenomena arise *via* intrinsic self-organization tendencies^2^ (Rietveld et al., 2018). Therefore, *deterministic* predictions or casual insinuations are replaced by *indeterminate* interventions, more productively considered as *system probes*. Due to the inherent, ecological complexity of a system, a probe may or may not initiate the change intended, meaning one cannot impose a specific course of action, only probe, sense, and then respond (Snowden and Boone, 2007). Contrary to traditional beliefs regarding human agency and intentionality (for a critique see Juarrero, 1999), *submission to constraint leads, and perceptual mastery adaptatively follows* (Woods, 2021).

However, a distinctive feature of transdisciplinarity is weaving lines of inquiry that may have remained isolated from one another due to disciplinary traditions and perceived boundaries (Woods et al., 2021a). In that spirit, we find a generative strand of conceptual alignment between socio-cognitive and ecological notions of values. Specifically, we note that values are constitutively related in a systems-oriented dynamic (Hodges and Baron, 1992; Maio et al., 2009; Schwartz et al., 2012; Borg et al., 2017; Hodges and Raczaszek-Leonardi, 2021).

Basic human values have been shown to exhibit dynamics along a circular continuum (Schwartz et al., 2012), whereby adjacent values amplify and/or “bleed over” into one another, while diametrically-opposite values exhibit a trade-off or “seesaw effect.” For example, an emphasis on social status can bleed over and amplify individual achievement, while simultaneously dampening universalism and tolerance (Maio et al., 2009). Our ecological account of these dynamics is detailed by Vaughan et al. (2021) and outlines how football environments that emphasize social status (power) may shape a value-directedness toward affordances that can partially realize that value (Hodges and Baron, 1992). For example, in a specific football performance context, a player's attention might be value-directed toward a dubious affordance to shoot at goal, rather than an almost certain opportunity to provide an assist for a teammate to score. In this example, our ecological conception of the “seesaw effect” is one whereby the value directedness experienced shines a bright light on affordances for “personal glory,” at the expense of, and overshadowing, affordances that are value-directed toward “collective collaboration” (e.g., passing to provide an assist) (Vaughan et al., 2021). Equally, the same value-directedness experienced in defensive moments may shine a light on affordances for heroic individual defending (e.g., a “last-ditch” tackle), rather than a more mundane, yet highly important, collective synchrony of movements to deny time and space.

Crucially, we contend that the value-directedness of intentionality experienced in any football environment is related to, and/or “weighted by,” the sociocultural significance entangled in forms of life and the broader ecology (see Vaughan et al., 2021 for detailed descriptions). In this case study, we aim to illustrate this “weighting” by using an adapted values continuum (from Schwartz et al., 2012) as a signification landscape. We contend that ethnographic themes overlayed on the values continuum can illustrate a value-directedness cascading into football environments and shaping player-environment intentionality (Vaughan et al., 2021).

## Football in Stockholm: the Present Study

From a transdisciplinary point of departure, sport organizations (guided by a Department of Methodology) are encouraged to embark on a journey to first establish, and then harness, their form of life (Rothwell et al., 2020). The following case study exemplifies the start of one such journey, outlining an attempt to more fully and faithfully understand the form of life coupled to an ATDE in Stockholm, Sweden. Understanding the form of life within the framework of the SIF gives insight into why athletes selectively engage with some affordances, and not others (Vaughan et al., 2021). In this study, we aim to characterize the interactions of people and places within a form of life and demonstrate that value-directedness can be illuminated *via* ethnographic endeavor (O'Sullivan et al., 2021).

### The Epistemology of Ethnography

Incorporating ethnographic endeavor within the frameworks introduced thus far, requires that we foreground an important epistemological distinction between (indirect) knowledge *about* and (direct) knowledge *of* the environment (Gibson, 1979; van der Kamp et al., 2019). Ethnographic endeavor is predominantly documentary, founded on the collection of data (audio, video, written field notes) relating to second-hand knowledge *about* the environment. Comparatively, the value-directedness of player-environment intentionality constitutes a responsiveness to affordances simultaneously arising from knowledge *of*, or direct perception *in*, the environment (van Dijk and Rietveld, 2017; Vaughan et al., 2021). Elaborating on this direct and indirect distinction of knowledge, Gibson (1979) explained that “[d]irect perception is what one gets from seeing Niagara Falls, say, as distinguished from seeing a picture of it. The latter kind of perception is mediated” (p. 139). In other words, “images, pictures, and written-on surfaces afford a special kind of knowledge [*about* the environment] … mediated or indirect, knowledge at second hand” (Gibson, 1979 p. 42; text in brackets added). It has to be noted that knowledge *about* the environment is associated with the traditional modes of communication used by coaches, trainers, and practitioners in most sports organizations. Thus, coach education programmes are deeply infused with pedagogical methodologies dominated by the transmission of abstract knowledge *about* the performance environment (Vaughan et al., 2021; Woods et al., 2021a).

Central to this study, Araújo et al. (2017) stated that gains in direct perception (e.g., gaining knowledge “of” one's environment) may be mediated through communication *about* the environment. While communication *about* (i.e., “talking about”) the environment is far from optimal for learners/performers (who require direct knowledge *of* affordances in the game to enhance their perception and actions), it can be useful in uncovering broad sociocultural constraints (e.g., ACC/neoliberalism) that influence the value-directedness of player-environment intentionality. We contend that, if gains in direct perception can be mediated through second-hand knowledge *about* the environment, then second-hand information that is collected *via* ethnographic endeavor and used to create themes in this study can serve to illuminate the sociocultural constraints, that shape the value-directedness of player-environment intentionality which, in turn, frames the perception-action couplings for affordance utilization in team sports like football.

## Methods

### Ethnography and Ecological Psychology

van Dijk and Rietveld (2017) suggested that studies in ecological psychology would benefit from ethnographic methods that themed the patterned practices of a form of life in an organization or community. They stated that in order to understand how “we respond to affordances offered by the material aspects of the environment and by other people, it is crucial that we understand the practical situation in which behavior occurs” (van Dijk and Rietveld, 2017, p. 2). When detailing the value-realization of affordances, Hodges and Baron (1992) have suggested that artifacts and actions in physical, social, and cultural settings constitute values and may “speak” as loudly, if not louder, than any words about such matters. The ethnographic case study employed here allowed a rich exploration of the extent to which social and cultural patterns of life are embodied in the way football is played and skills developed (van Dijk and Rietveld, 2017; Vaughan et al., 2021).

### Ethnographic Case Study

In 2017, the first author commenced an 18-month ethnographic study embedded in AIK football club. He was involved in the club's day-to-day activities as an academy, school, extra training, and holiday camp coach. Off the pitch, he assumed the role of research co-ordinator. Further, the first author actively immersed himself within the day-to-day life of the city and its people (LeCompte and Schensul, 1999). Participant observation—a process of data collection through exposure to, or involvement in, the day-to-day, routine activities in the research setting—was undertaken. It is an approach to data collection that requires the researcher to be present at, involved in, and actually recording the routine daily activities of people in the field setting (Schensul and LeCompte, 2012). However, the role of observer is not static, but a continuum from participant-as-observer (someone who participates in activities, but not in everything) to observer-as-participant (someone who participates moderately, but primarily observes from the sidelines) (Ives et al., 2019).

Participant observation ethnography does not require a specified group of participants (Schensul and LeCompte, 2012), especially when behavior is observed in public settings—like the sports grounds of Stockholm. These public observations pose no threat or consequence either to the observer or the observed (Schensul and LeCompte, 2012) and people are not identifiable within the data (Tracy, 2013). However, participant consent was sought from the youth club's senior leadership group and key day-to-day club colleagues, whose voices are represented (with pseudonyms) in the data. Participant consent forms were signed and secured in the club's head office. Prior to data collection for this study institutional ethical approval was granted by The University of Queensland, approval number HMS16/27/09.

Four hundred and 25 field notes of varying media and length were generated from ~2,520 h of immersion in the field. Opportunities for data collection (in particular, informal conversations), occurred in unpredictable ways, at unpredictable times and, therefore, the lead author was never really “off duty.” The note keeping and categorization application Evernote, was used to collect, sync (across phone and computer) and organize field notes in the form of audio, video, image, and text files. Field notes varied from *in-situ* audio recordings (of meetings and informal conversations^3^) and videos of training and games, to post observation head notes, which incorporate the thoughts and feelings of the first author (Tracy, 2013). National media and sport/football/club documents were also analyzed, and data were predominantly collected in Stockholm from March 2017, until June 2018. During this period a regular, in-season coaching week included three academy sessions, two extra training sessions, one school session, and, often, two games at the weekend. The first author also coached daily at five holidays camps, with each camp lasting four days, running from 9 am to 3 pm. Data were also collected at a variety of organized meetings, events, (sport governing body) conferences, senior football matches and educational evenings (e.g., parent seminars and coach education courses). Ethnographic immersion and data collection took precedence for around 18 months, at which time it was decided that a (data collection) saturation threshold emerged.

### Data Analysis

*Phronetic iterative qualitative data analysis* was conducted (Tracy, 2018). This iterative analysis alternated between emic (or emergent) readings of the data and an etic use of existing models, explanations, and theories (Tracy, 2013). Analysis alternated between: (1) data generation, (2) scrutinizing emergent findings from the data, and (3), consulting existing theoretical and conceptual frameworks underpinning this case (Tracy, 2018). The ethnographic research process allowed data analysis to commence with data generation (LeCompte and Schensul, 1999) beginning with the elaboration and transcription of raw field notes (Tracy, 2013). The writing of formal field notes enabled us to *scrutinize emergent findings*, as ethnographic data were read, documented, elaborated, grouped, and categorized into coherences, or themes. The emic emergence of themes is illustrated in a 18,672-word *results narrative* that serves to simultaneously analyse, synthesize and further scrutinize and contextualize themes. Richardson (2000) has stated that such writing is a method of inquiry, whereby researchers analyse the subject of interest and simultaneously acknowledge their interpretive stance (which is what others have referred to as an expression of “researcher personality”; Matapo and Baice, 2020). Several strategies were used to promote analytical rigor, guided by ideas of Tracy (2010) and Smith and McGannon (2018). These strategies are exemplified by: a) appropriate use of data collection and analytical procedures, b) attempts to be sincere to the data and challenge potential subjective biases, and c), the use of a critical friend (second author), who is highly experienced in qualitative research to challenge the interpretations (Smith et al., 2014).

## Results and Discussion

### Emergent Findings

**Figure 1:**
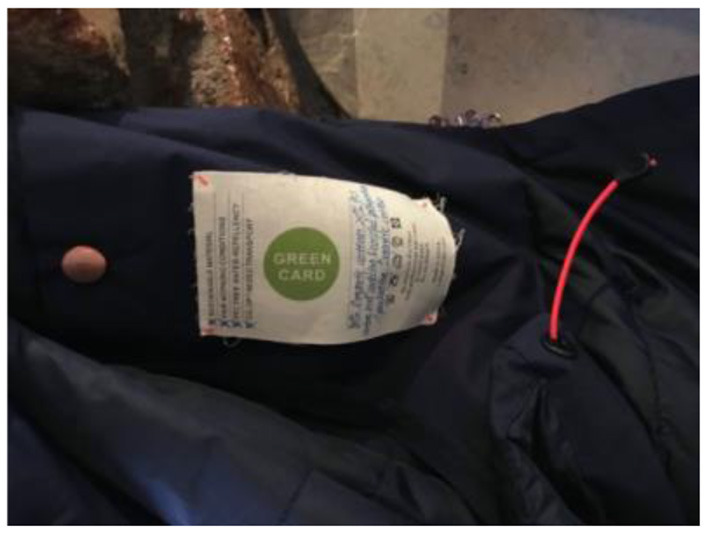
The green card inside my winter coat.

**Figure 2:**
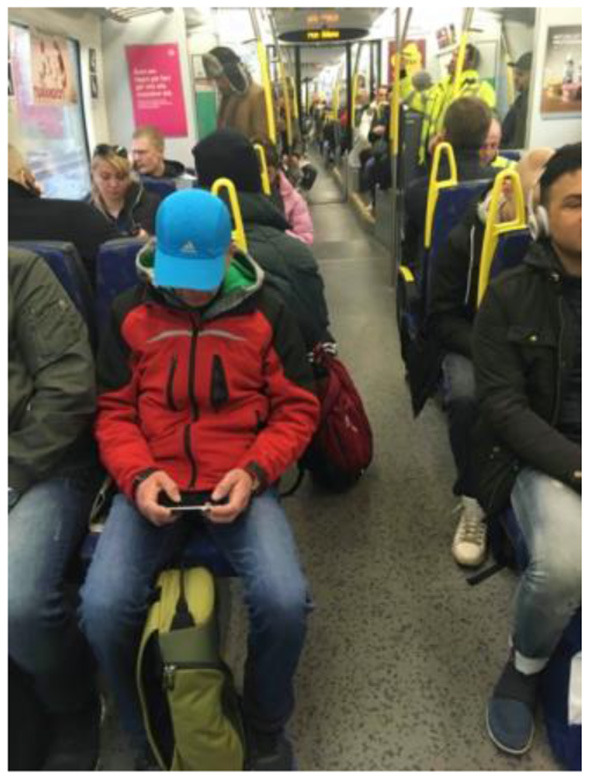
Train at 6.07 am.

Results of the case study are storied, in which text and images (data) are integrated in a way that contextualizes and foreshadows the coherence of themes. Portraying the day-to-day experiences of a football coach in Stockholm, the fact-based fiction charts the daily transition from the macro environments surrounding the football club, to micro-environments within the club (describing on pitch observations and coaching experiences). The story covers a 12-h period and begins with the commute to work, highlighting the everyday familiar sights, sound, and artifacts of the macro environment, before moving onto the specifics of football development microenvironments. Reflecting on the everyday artifacts and routine experiences, the storyteller introduces themes emerging over the course of the study. While presentation of the full narrative (provided as Supplementary Material) is beyond our scope, representative fragments are presented (single spaced), with raw data (text) included as quotes and or imagery (see Figures 1, 2)—for example:

I step out of the HSB apartment building just before 6:00am. The air is dry and very cold, it reminds me of last night; minus 9 is (great for skiing but it's) not ideal for playing (or coaching) football. It's dark, and as I walk toward the train, my feet crunch the refrozen remnants of yesterday's snowfall. I'm thankful for my new winter coat, a combination of practical warmth and sleek Scandinavian design.

Designed to combat climate change and sooth the conscience, the coat contains a “Green Card” stating the manufacturer's use of sustainable material, fair working conditions, PFC free waterproofing and CO_2_ optimized transport. Stockholm is described as “a city for the conscious middle class and for the rich” (Document analysis: Svenska Dagbladet newspaper article, 21st August 2017): a city where people condemn climate change and plan exotic, CO_2_ emitting, vacations in the same breath.

However, winter is awash with refined dark coats and concerned looks (perhaps that's just the temperature) and while the hot pink elastic toggles (on my coat) cut through some of this monotony, they are only a minor divergence from the norm—helping to strike some balance between “fitting in with” and “standing out from” the crowd: A modern example of the Swedish value in lagom perhaps.

“Lagom is often translated into English as “not too much, not too little, just right.” It's a word that implies understanding what the extremes are and finding the moderate path in between the two. It means behaving appropriately, eating the right amount, and on the flip side, celebrating the right amount too. In Swedish it's an all-encompassing word, one that can be directly applied to almost every element of society. Lagom food, lagom drink, lagom work hours; the idea that a balanced amount of everything leads to a just and equal society and, overall, a good life.” (Document analysis: Live Lagom, Balancing living the Swedish way, Brones, 2017).

Lagom work hours would be nice; I step onto the 06:07 train and I can't find a seat. This morning I have a 07:00 meeting with AIK's head coach and his staff at Karlberg… I customarily check my phone and see an email from Saturday night, sent at 23.28 (by an AIK colleague). “We don't work full time, we work all the time” is the motto fondly (sometimes exasperatedly) attributed to the youth club's sporting director, who has been active in the club for 54 years.

**Figure 3:**
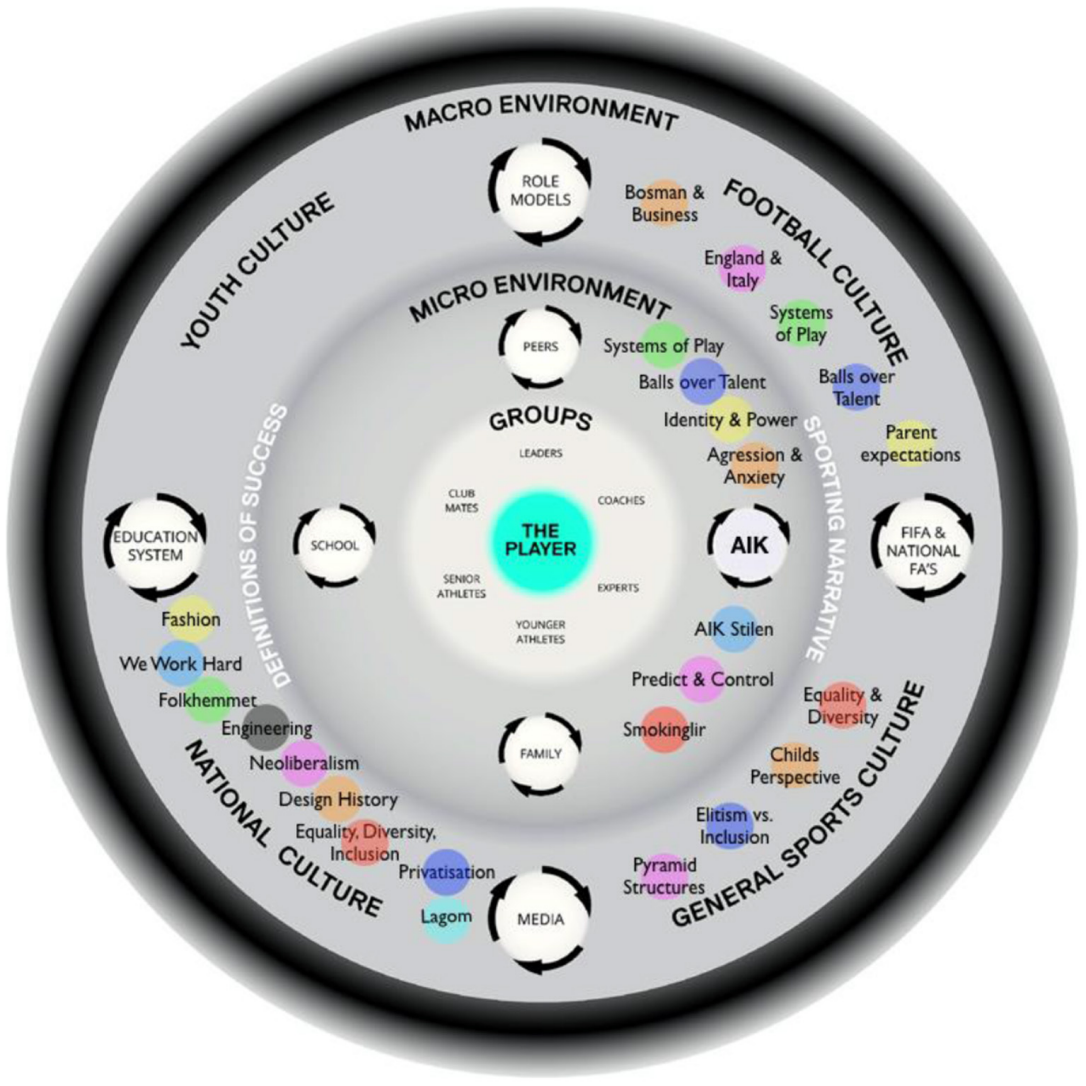
The football in Stockholm ATDE. Themes are embedded in relation to the environment/context (macro or micro) in which the data emerged and cohered. Colors relate to placement on values continuums in later Figures.

**Figure 4:**
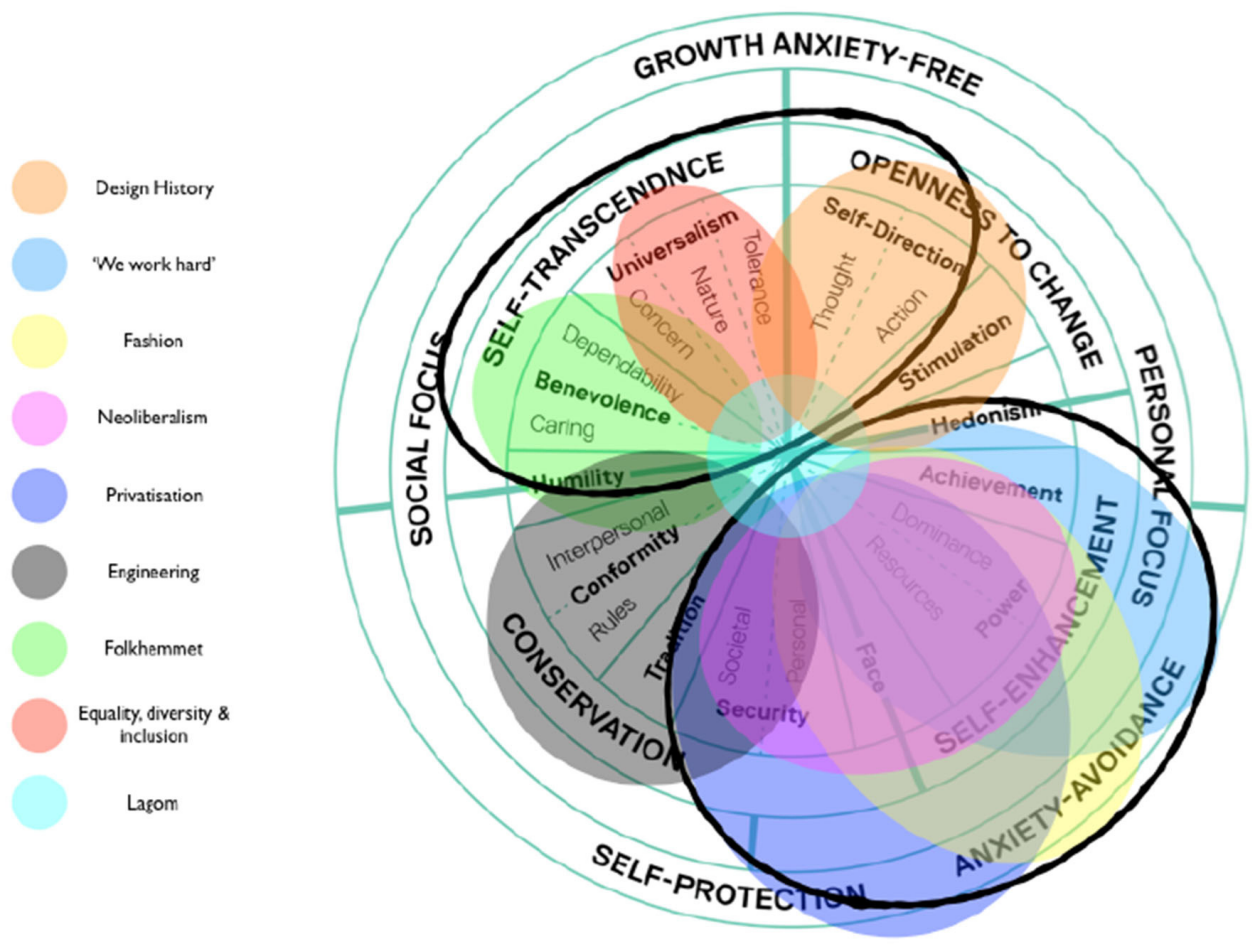
An illustration of value-directedness of themes relating to Swedish national culture. Value-directedness weighted toward competition (bottom right) and away from collaboration (top left) at the level of Swedish national culture illustrated by emergent themes in the macro environment. Adapted from Schwartz et al. (2012).

**Figure 5:**
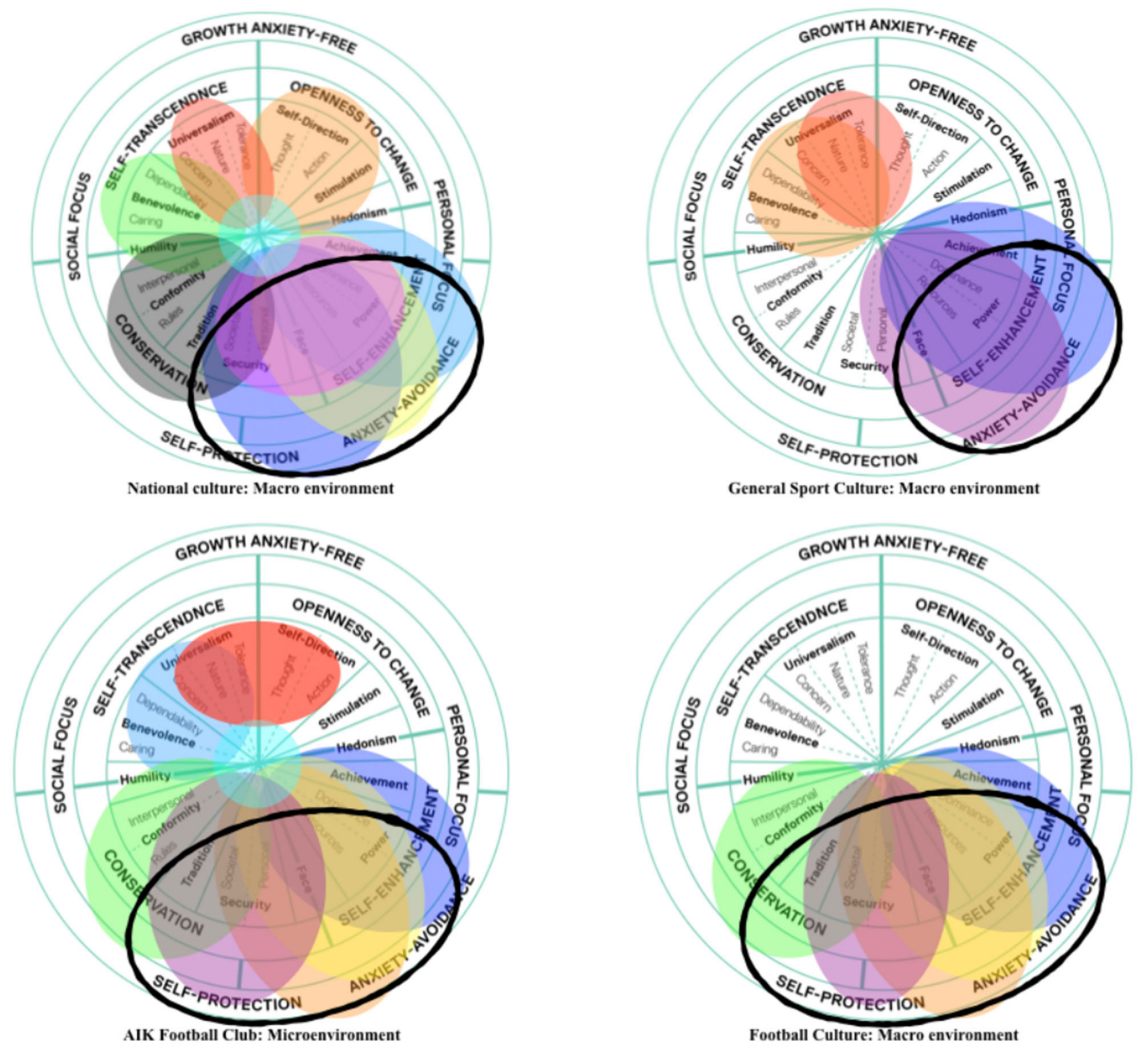
The value directedness of cultural sub-systems at embedded levels of the Football in Stockholm ATDE. Value-directedness weighted toward interpersonal competition (bottom right) and away from collective collaboration (top left) across sub-cultures and contexts relevant for athlete development within the ATDE.

Data are presented as such to reveal the contextualized meanings of themes (e.g., ‘lagom' and ‘we work hard' above) and inherent tensions (contradictions) between themes. Utilizing the terminology of Schein (1990), the narrative extract above demonstrates how the espoused value in lagom (balanced and acceptable) work hours sits in tension with, and is overshadowed by, the underlying assumption that “we work all the time.” Data presented in narrative form demonstrate the inherent relations and tensions between themes, supporting Schein's, 1990) proposal of culture as an interconnected and embedded ecology of systems striving for equilibrium (Schein, 1990). However, the data also illuminate an important qualification around cultural equilibrium, being that “systems contain subsystems, organizations contain groups and units within them, and it is not clear over what range the tendency toward equilibrium will exist in any given complex total system” (Schein, 1990, p. 110). Hence, it is recognized that sub-systems often exist in quasi-stable, non-equilibrium states (Graedel and Allenby, 2010). To concretize these dynamics (complex tensions and trade-offs) inherent in Swedish culture as they relate to athlete development, etic analysis was employed to delineate themes upon an ATDE model (see Figure 3) and reveal the value-directedness of themes in specific cultural contexts using an adapted values continuum model (see Figures 4, 5).

### Etic Use of Existing Models, Explanations, and Theories

An ATDE (Henriksen et al., 2010) was used as a formative model for data collection, organization and presentation of themes (embedded in Figure 3). To illustrate the cascading influence of (value-directed) themes throughout cultural sub-systems and into the embedded levels of AIK's athlete development environments, themes were also embedded on a values continuum (see Figure 4, the colors of themes are the same across all Figures). Placed on a values continuum, the “weight” of themes can be illustrated (signified) as they overlap and coalesce around particular clusters of values. Collectively, the image created (e.g., Figure 4) represents a system of value prioritization, and the weight (i.e., overlap of themes) illustrates the value-directedness of intentionality, that may be experienced by young football players at AIK.

Figure 4 shows themes related to Sweden's national culture overlayed on a values continuum and illustrates a weighty cluster of themes (‘We work hard', ‘Fashion', ‘Neoliberalism' and ‘Privatization') that overlap around the value in face (prestige/social status) and power (dominance over people and resources). The two clusters of themes circled in Figure 4 illuminate a meta theme, highlighting the tension between collective collaboration and individualistic competition. Most themes served to emphasize one or the other, however, as displayed in Table 1 and evident on Figure 4, the value-directedness emphasizes *individualistic competition over collective collaboration*, which appears as a fractal pattern (at many dimensions) across embedded sub-systems in an environment, see Figure 5. In Figure 5, the quasi-stable, non-equilibrium dynamics of cultural sub-systems are evident (Schein, 1990), with football culture displaying a state of non-equilibrium, heavily weighted by themes that emphasize a value-directedness toward individualistic competition. When value-directedness is experienced as rigid and inflexible, as illustrated at the level of football culture, we contend that it characterizes a sociocultural constraint that shapes forms of life (Vaughan et al., 2021).

**Table 1:** Key themes arising from ethnographic data under the meta theme individualistic competition over collective collaboration.

**Competition**		**Collaboration**	
**Meta theme: individualistic competition over collective collaboration**			
Themes directed toward competition	Related themes	Theme directed toward collaboration	Related themes
**Macro environment: Swedish national culture**			
1. “We work hard”		1. Design (history)	
2. Privatization		2. Folkhemmet	
3. Fashion		3. Equality, diversity and inclusivity	
4. Design (current)			
**Macro environment: Swedish Sport**			
5. Elitism over inclusion	12. Pyramid structures	4. Equality and Diversity	7. Child's perspective
**Macro environment: Swedish Football**			
6. Bosman and Business	13. England and Italy		
7. Parents' expectations	14. Systems of play		
8. “Balls over talent”			
**Microenvironment: AIK Football Club**			
9. “Balls over talent”	15. Predict and control	5. Smokinglir	
10. Identity and Power	16. Playing style	6. AIK Stilen	
11. Anxiety and Aggression			

We surmise the sociocultural constraint of value-directedness as an intentionality directed toward (preferencing) opportunities for individualistic competition, specifically affordances that protect or enhance social status. We propose that this rigid value-directedness can confine perceptual attunement to a limited range of properties in an environment, with fewer soliciting affordances standing out. Under these circumstances, we characterize the intentionality experienced, and the emergent footballing form of life as *unskilled* (Vaughan et al., 2021).

### Anxiety and Aggression

The following section of narrative presents data relating to the value directedness represented in Figure 5 and relating to AIK's microenvironment as well as the broader football culture in Stockholm. Data relates to the theme “Anxiety and Aggression”:

I'm observing the 07 group (players born in 2007); the intensity is frantic as tackles fly in left, right, and center. However, these periods of ferocity are punctuated as players break into tears. More often than not the pain isn't physical, but mistakes bring gasping sobs and injuries are feigned to cover over the emotional turmoil. I turn to Matthias and ask if this is normal, he nods… It's hard not to be impressed by the speed and skill of the individual play, but it's equally hard not to despair that kids of 10 years old are feigning injury and crying because they've made a meaningless mistake… It's obviously not meaningless to them; again, I'm left wondering why?

After we've packed up and said our good-byes to the kids, I'm standing with Ben (07's coach) and watching another session when Mike approaches. Mike is Nick's Dad; Nick is one of the most skilful players in the 07 group and he's also, often, bursting into tears. Mike is always dressed in fresh, fashionable athletic apparel, like the limited addition stuff sports brands make, his trainers always seem brand new, and his hair is slicked back. I've been told he's a CEO of a company. He's not unfriendly, however when I arrived at the club, he quizzed me, asking that I compare the 10-year-olds at FC Barcelona with his son's group. Today, he talks quietly as we discuss the competition in the team and the decision to increase the age of the academy intake (from 8 to 13).

Mike: I think the atmosphere can be quite tough sometimes I hear, Nick tells me…. they are nice to each other, but they can all be tough to each other, because they are like Alphas, they are winners they wanna be tough guys, and I'm the next Messi, and sometimes that has an impact on the atmosphere, but I think that's also life.

(Field note: Informal conversation, May 14th, 2017).

Comparison and internal competition are themes I'm familiar with, a conversation with a friend at the English Institute of Sport comes to mind:

Matt: It's my biggest concern as a parent (in sport) and you can see it with Charlie… you can see it everywhere… the status anxiety or social judgments continually going on, you know kids are continually comparing themselves to each other, and yeah (sighs)… it just shuts so much down, it's so unhealthy but it's so hard to… you know… it's a classic again because you can't have that conversation with a 9 year old, so you've got to shape a different environment, you can't explain it rationally.

(Field note: Informal conversation, November 17th, 2017).

Talking to Mike by the side of the pitch I try to suggest that maybe it doesn't have to be the way it is?

James: I think that's what this new structure in the academy is about… Maybe it doesn't need to be like that at 10 years old… I mean you look at some of the best players… some of the most intelligent players, like the Xavi's, Iniesta's and Messi's they're not necessarily Alphas.

Mike: No, they are pretty low-key guys.

James: No one wants to lose, everyone wants to play the game, and be competitive and win… but it also doesn't matter enough that your coming off crying… because that's a learning opportunity and that's what I worry about, where is that pressure coming from?

Mike: Well, that's coming partly from the parents… definitely…

Ben: And the culture around the academy…

Mike: I think there is a problem, like with us parents of course there's an expectation that the team should always win… of course there is… then of course… not always win, but I mean when you lose a game 5-0 to Nakdala all the parents are like, okay what the hell is happening? People say, “we're an academy, we have such great players how we can lose to Nackdala 5-0,” when we know that we could beat them 8-0 the next day if we just have our head screwed on.

(Field note: Informal conversation, May 14th, 2017).

I recall the game in question and experience the hollow feeling accompanying the memory. I remember feeling “the mood is so serious and so toxic even I feel “bad” standing on the sideline” (Field note, 29^th^ April 2017). At half time, we had players in tears and during the second half we had to substitute one of our players as he aggressively, continuously, and violently fouled (and hurt) opposition players. After leaving one opponent gasping for breath, our player refused to apologize saying the Swedish version of: “I don't give a crap” (Field note, 29th April 2017). At the end of the game our captain ripped the armband from his shoulder and winding up like a baseball pitcher threw it to the ground before storming off in tears. At the end of the game, players either sobbed on the ground or stormed around kicking, screaming, and swearing. On the train home I remember feeling shaken… I had to ring a friend to clear my head… “this isn't a healthy environment for players or coaches” (Field note, 29th April 2017). I'd heard Pontus and Stephen talking about issues with performance anxiety… but to witness it in such a tangible way was horrible. It reminded me of Johan Fallby's (Sport Psychologist) talk at the Stockholm Sports Federation's Conference.

Fallby said: I've been in junior teams' dressing rooms that are more filled with anxiety than the first team before the Champions League play-offs (at FC Copenhagen).

(Field note: Headnote 29th April 2017).

Even away from academy duties these (controlling) behaviors seem to persist in some academy players. At a recent holiday program, three academy players oscillated between tearful tantrums and authoritarian outbursts.

The captains of their respective teams and the oldest players at the camp, the behavior of the academy players became more extreme and embarrassing as the morning wore on. They verbally abused the younger kids in their team before breaking down into tears if they lost a game. At one point a 7-year-old boy left the pitch in tears after an eleven-year-old academy player screamed at him after the final whistle—they were supposed to be teammates.

(Field note: Headnote: 6th April 2018).

At 7 years old this kid was humiliated, shamed, and initiated into the world of football by an academy player he probably looked up to. I recall Stephen telling me that the average age for kids to drop out of football has fallen to 9 years old in the north of Stockholm. I can see why.

The evidence moves us to propose that observations of anxious and aggressive interactions in and around football emerge from a value-directedness toward interpersonal competition. Adopting a Bourdieusian metaphor this can be portrayed as a “game” within a “game” (for a detailed training session example see Vaughan et al., 2021). The first game explicitly being football, the second implicitly embedded, competitive game being to gain, maintain, or protect social status (capital) within the game of football. Supporting this explicit-implicit synthesis, adolescent aggression has been positively correlated with self-enhancement values (Benish-Weisman, 2015), while competing for social status has been identified as motivating aggression (Salmivalli, 2010). In football coaching, expressions of self-protection, anxiety, manipulation, aggression and controlling behaviors are also evident (Potrac et al., 2013); exemplified in Stockholm *via* document analysis relating to the theme “balls over talent” (see Figure 5):

It was Aftonbladet who revealed, on Friday afternoon that Broma boys' coach Luis Pimenta controls the team through threats, harassment and pure bullying. Several players anonymously described a leadership based on a macho culture that violates everything Brommapojkarna stands for, as one of the country's largest youth clubs.

After the big loss against Häcken away of 0-6, Pimenta has forced the players to stand up in front of the others and talk about situations in the match when they played like a “pussy” and when playing with “balls.” Throughout the season, Pimenta has also had a hierarchy list on the wall where he ranks the various players.

(Document analysis: Dagens Nyheter article: Efter anklagelserna: BP:s tränare tvingas ta time out, September 2018. Translated from Swedish)

A similar trend is evident at broader macro levels beyond Stockholm and Sweden. In his article titled *What's wrong with Argentina? We now value “balls” more than talent*, the ex- Argentinian professional and World Cup winner, Jorge Valdano, describes a situation whereby the game of football has become secondary to other events:

“At the same time a passion for football was overcome by a passion for a team, as if a society that has become ever more individualistic needed something to reconnect it with tribal feeling. Turning clubs into mini nations constructs an identity, a community that must be defended as a matter of life and death. In the stands violence took over; on the pitch, we said goodbye to the olés and welcomed in a world where huevos—balls—are more important than talent.”

(Document analysis: Guardian article: What's wrong with Argentina? We now value “balls” more than talent, 25th June 2018.).

**Figure 6:**
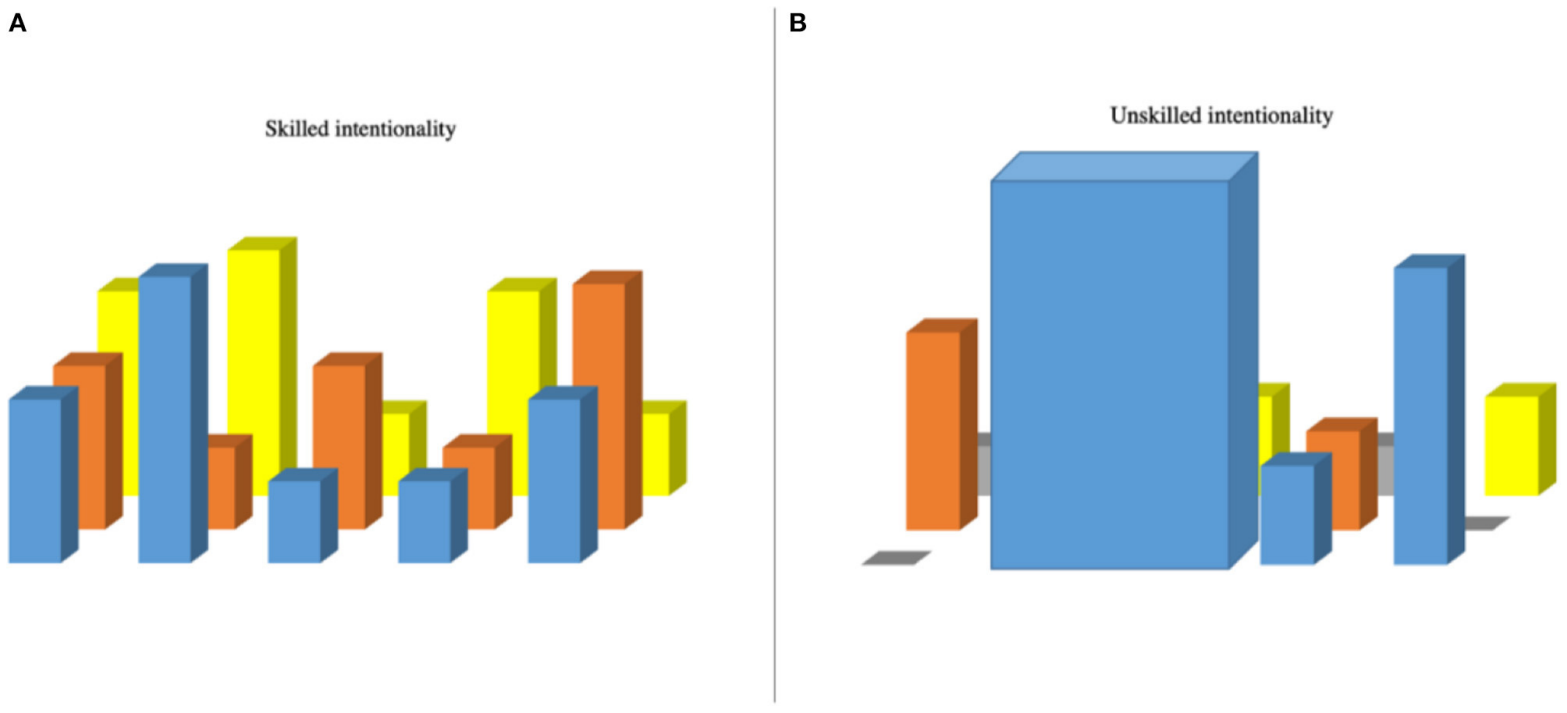
A field of relevant affordance shaped by skilled **(A)** and unskilled **(B)** intentionality. Figure illustrates **(A)** an athlete's responsiveness to a field of affordances indicative of skilled intentionality; and **(B)** illustrates a field of affordances indicative of unskilled intentionality, whereby an overbearing value-directedness leads some more soliciting affordances to “stand out,” while others become overshadowed, and more still are imperceptible (gray).

Schwartz et al. (2012) theorized that a value-directedness toward the lower half of the values continuum directs people toward protecting themselves “against anxiety and [psychological] threat” (p. 668). In our observations, this value directedness can be characterized by a fear of making mistakes and displays of performance anxiety in football. We suggest that such value-directedness is experienced as a sociocultural constraint when a responsiveness to soliciting affordances (e.g., those that are “safe” from negative social judgement) overshadows others and narrows the field of perceptible affordances^4^ (see Figure 6, inspired by Rietveld and Kiverstein, 2014).

When responsive to only a narrow range of affordances we propose that players and teams are displaying *unskilled intentionality*, meaning they are unresponsive to certain affordances (e.g., shared, and nested affordances) within the footballing form of life. In this case, the unskilled intentionality experienced results in responsiveness to affordances that are “safe” from negative social judgement. Empirical observations suggest that unskilled intentionality results in a way of playing football that is characterized by one-directional (linear), predictable, and rigidly patterned play devoid of adaptive solutions, moments of creativity and markers of skilled intentionality (see Vaughan et al., 2019, 2021).

### Unskilled Intentionality: A Football Form of Life Unresponsive to Nested Affordances

Unskilled intentionality was empirically evident in football (matches and training) that appeared one-directional, predictable, and rigidly patterned. This is exemplified in a short fragment of the results narrative below (in italics):

The memories of my first AIK holiday program flood my mind; I was coaching with Jake and we were shocked by the linear pattern of play during the matches. Eleven and twelve-year-old players clustered around the ball like bees around honey, seemingly obsessed with only moving forward. If a player happened upon the ball, they plowed forward making a beeline for goal with little appreciation of wide spaces or gaps between opposition. Depending upon the manner in which the ball arrived, the forward motion manifested as a “big kick” or often a bumbling, stumbling dribble. Like an endangered species, passing was scarce. The game took on a formulaic tendency; a linear pattern of dribble forward…get tackled, fight for the ball, lose the ball and then the opposition repeats the linear method; dribble forward, get tackled, lose the ball and repeat and repeat and repeat. It was almost as if the players had blinkers on: Attention attracted by ball and goal, nothing else.

This one-directional focus also became increasingly obvious at a recent academy Under 10 tournament in Berlin. As the games progressed and the intent on winning intensified, AIK's young players seemed to suffer from an increasingly severe case of tunnel (reduced) vision. Almost every AIK throw-in was hurled forward into a pack of players thrashing around like piranha. I remember AIK's head of academy turning to me on the sideline and questioning why “they never throw it back to the goalkeeper?,” the player with the most time and space.

Any intention to utilize time and space—the emphasis of many training sessions during my time in the academy with this team—evaporated as the ball was feverishly booted, or thrown, forward to cheers from parents. Our players started pulling opposition shirts and throwing arms and elbows, some tackles started to resemble rugby more than football. At this point, even if players were brave enough to try and pass or dribble, they often had no-one to pass too as their teammates were hiding, seemingly anxious of making a mistake.

(Field note: informal conversation, 15th September 2017).

### Practical Considerations

Our observations in this case study illustrate the extent to which the value-directedness experienced might foster unskilled intentionality in football. Football coaches and parents of very young players will recall an obvious example of unskilled intentionality, characterized by the type of swarming football classically referred to as players following the ball, looking like “bees around honey” (see Vaughan et al., 2021). In this example, (player-environment) intentionality appears to emerge as a directedness toward *the ball* (regardless of available affordances, such as, space, location of teammates, opposition or goals). By guiding attention toward direct interactions with the ball, this unskilled intentionality creates the swarming (phenomena) reminiscent of “bees around honey.”

From this reference point, football player development requires the education of attention beyond the ball and goals, and toward other perceptual information (location of teammates, opposition, gaps, spaces) and the rich field of affordances (Rietveld and Kiverstein, 2014) in football's form of life (Vaughan et al., 2021). As such, the role of club personnel, including coaches and practitioners, is to develop, substantiate and work within (and understand) their form of life to shape player-environment intentionality that is progressively skilled. We propose that a key aspect of fostering skilled intentionality is appreciating the sociocultural constraints and associated value directedness resonating within one's form of life. Once identified, we contend that sociocultural constraints might be amplified or dampened by re-shaping the value-directedness of player-environment intentionality toward optimal relations (i.e., affordance utilization) that enhance skill development (Bruineberg and Rietveld, 2014; Vaughan et al., 2019, 2021). While a full account of practical applications for coaches and clubs is beyond the scope of this paper, we provide evidence for the need to *shape (the value-directedness of) players intentions* in order to frame perception-action cycles and reveal the rich landscape of affordances (and related effectivities/skill sets) to be discovered in football player development (Rietveld and Kiverstein, 2014; Rasmussen et al., 2017; Vaughan et al., 2021). We view *shaping player's intentions* as an additional principle of non-linear pedagogy (Vaughan et al., 2021; Chow et al., 2022).

## Strengths and Limitations

An important strength of the ethnographic approach and broader methodology adopted here concerns the collection of information on culturally-embedded perceptions and lived experiences that are open to analysis and synthesis *via* transdisciplinary lenses and iterative data analysis (Tracy, 2013). Embarking on ethnography, as a cultural outsider, brings strengths and weaknesses to the work. While the outsider perspective can be at odds with having a fluent grasp of nuances in the local language, it ensures that processes of enculturation or acculturation have not obscured one's sensitivity to underlying, and potentially significant, cultural assumptions (Vaughan, 2020). In this study, interactions with helpful locals (explaining or translating a colloquial expression or nuanced cultural perspective) often led to conversations that surfaced deep reflections and provided opportunities to uncover basic underlying assumptions (Schein, 1990). In surfacing culturally embedded perceptions and the lived experiences of athletes, ethnographic inquiry is particularly relevant for sports coaches and associated practitioners in day-to-day interactions with groups of athletes.

The ecological rationale we present makes it possible to transcend traditional perspectives on (*a priori*) values and skills as internalized representations within individuals (Rothwell et al., 2018, 2019). It illuminates the extent to which the emergence of skill (Hristovski et al., 2012) is irreducibly entangled with the value-directedness of player-environment intentionality, an aspect of the whole ecology of relations or form of life (van Dijk and Rietveld, 2017; Vaughan et al., 2019, 2021; Vaughan, 2020). Nevertheless, it is important to note that the positioning of themes on a values continuum (e.g., Figure 2) is a visual aid to represent a “weight” of sociocultural significance and value-directedness. The positioning is not exact because the values (and therefore themes) themselves displayed on such a continuum exhibit “fuzzy” boundaries (Schwartz, 2012), indeed values bleed over and into, and strengthen one another (Maio et al., 2009). Therefore, the positioning of themes is not intended to be calibrated with precision. Inferring such a level of exactness in reported data would be misleading, given the qualitative nature of this study and the quasi-stable/non-equilibrium states of sociocultural systems under investigation (Graedel and Allenby, 2010).

## Conclusion

This study suggested how the values that athletes embody are *constrained* by the *character* of the social institutions (sport club, governing body) and the social order (culture) in which they live. We framed this *constraining character* using the nested concepts of a form of life, sociocultural constraints, value-directedness, and relevant fields of affordances. Empirical evidence from the 18-month ethnographic case study highlights how social and cultural constraints influence the skill development and psychological wellbeing of young football players. Empirically, this case study demonstrates that a sociocultural value-directedness toward *individual competition* overshadows opportunities (conceptualized as shared and nested affordances) for *collective collaboration* in football. These findings, therefore, characterize a sociocultural constraint that could limit the skill development (and performance) and psychological wellbeing of young football players in Stockholm. Perhaps this possibility is not surprising when we recognize the extent to which the microenvironments of athlete development are embedded within, and shaped by, broader cultural trends toward neoliberalism and competitive individualism in many societies.

A novel contribution to the literature is our illustration of how the value-directedness of intentionality can constitute a sociocultural constraint by shaping the fields of affordances that are perceived, and in turn, shape learning in development in football (Figure 6). Consistent with the theorizing of Hodges and Baron (1992), we found that dominant values can be observed *via* the artifacts and interactions of people framed by specific social and cultural settings. In this study, we found that an adapted values continuum was generative in explaining the value-directedness that characterizes the form of life in which football player development takes place. Utilizing ecological dynamics and the skilled intentionality framework this case study opens novel avenues for future investigations, and practical insights, related to constraint-led coaching (Renshaw and Chow, 2019) and nonlinear pedagogy (Chow et al., 2022).

## Data Availability Statement

The original contributions presented in the study are included in the article/Supplementary Material, further inquiries can be directed to the corresponding author/s.

## Ethics Statement

The studies involving human participants were reviewed and approved by School of Human Movement and Nutrition Sciences Ethics Committee, the University of Queensland. Approval number: HMS16/27/09. The participants provided their written informed consent to participate in this study.

## Author Contributions

JV, CM, and KD contributed to the conceptualization and background research. JV and CM developed the research design (data collection, organization, and analysis) and drafted the first version of the paper. KD, PP, CW, and MO'S made critical revisions to the first draft. All authors helped finalize and approve the paper.

## Conflict of Interest

JV is employed as Head of Development (13–19) AIK FC and is a co-founder of Player Development Project. MO'S is employed as Head of Development (8–12) AIK FC. The remaining authors declare that the research was conducted in the absence of any commercial or financial relationships that could be construed as a potential conflict of interest.

## Publisher's Note

All claims expressed in this article are solely those of the authors and do not necessarily represent those of their affiliated organizations, or those of the publisher, the editors and the reviewers. Any product that may be evaluated in this article, or claim that may be made by its manufacturer, is not guaranteed or endorsed by the publisher.

## Footnotes

^1^Following the recommendations of Sebbens et al. (2016) the term mental health problem is used to capture both mental illness (e.g., depression, anxiety) and symptoms of mental illness that may not be severe enough for a diagnosis. That is: clinical and sub-clinical levels of mental health challenges.

^2^Within the SIF, Rietveld et al. (2018) “see the emergence of the soliciting character of affordances as the result of a process of self-organization. Merleau-Ponty's (Merleau-Ponty, 1968/2003) philosophy of life helps us to see that the environment always already solicits something to the active individual. Merleau-Ponty observes that, as complex biological systems, living organisms are always simultaneously ‘in a state of relative equilibrium and in a state of disequilibrium' (p. 149). Crucially, this inherent disequilibrium “inspires or motivates self-organized compensatory activity” (Merleau-Ponty, 1968/2003, p.149; cited in Rietveld et al., 2018, p. 11).

^3^Initial one on one meetings were often akin to informal interviews, however, follow up conversations and interactions oscillated from practical meetings/follow up interviews and critical friend checks and balances Smith et al., 2014.

^4^Rietveld and Kiverstein (2014) illustrated a relevant field of affordances “along the following three dimensions: (a) broadness of scope of affordances engaged with (“width” of the field), (b) temporal horizon/anticipation (“depth”), and (c) relevance or solicitation of the perceived affordances (“height”).” (p. 348).

## Supplementary Material

Data Sheet 1 A Football Coach's view from Stockholm.
