# Supplementary material for: Social and Cultural Constraints on Football Player Development in Stockholm: Influencing Skill, Learning, and Wellbeing
Source: Front Sports Act Living. 2022 May 20;4:832111. doi: 10.3389/fspor.2022.832111 (PMC9163368; doi:10.3389/fspor.2022.832111)
Supplement: Data Sheet 1 — A Football Coach's view from Stockholm. [file Data_Sheet_1.pdf]

Supplementary Material: A Football Coach's View from Stockholm

Citation: Vaughan J, Mallett C J, Potrac P, Woods C, O'Sullivan M and Davids K (2022) Social and Cultural Constraints on Football Player Development in Stockholm: Influencing Skill, Learning, and Wellbeing. Front. Sports Act. Living 4:832111.doi: 10.3389/fspor.2022.832111

https://www.frontiersin.org/articles/10.3389/fspor.2022.832111/full

1.1 Lagom: Balance is everything?

I step out of the HSB apartment building just before 6:00am. The air is dry and very cold, it reminds me of last night; minus 9 is (great for skiing but it's) not ideal for playing (or coaching) football. It's dark, and as I walk towards the train, my feet crunch the refrozen remnants of yesterday's snowfall. I'm thankful for my new winter coat, a combination of practical warmth and sleek Scandinavian design.

Designed to combat climate change and sooth the conscience, the coat contains a "Green Card" stating the manufacturer's use of sustainable material, fair working conditions, PFC free waterproofing and CO_2 optimised transport. Stockholm is described as "a city for the conscious middle class and for the rich" (Document analysis: Svenska Dagbladet newspaper article, 21st August 2017): a city where people condemn climate change and plan exotic, CO_2 emitting, vacations in the same breath.

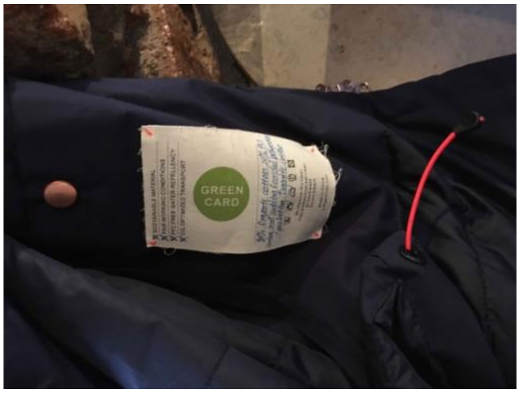

Figure 1. The Green Card inside my winter coat

However, winter is awash with refined dark coats and concerned looks (perhaps that's just the temperature) and while the hot pink elastic toggles (on my coat) cut through some of this monotony, they are only a minor divergence from the norm – helping to strike some balance between 'fitting in with' and 'standing out from' the crowd: A modern example of lagom perhaps?

If you ask Swedes about the history of lagom, many might begin with a story of Vikings. It's a romanticised version of lagom's roots, a story of the Vikings passing around a horn of mead. Laget om, or 'around the team', meant that the mead had to make it to everyone's lips, requiring that each Viking only take an adequate sip so that there would be some for everyone…

In Swedish lag means 'law' and in old Swedish, 'laguhm' translates roughly to 'according to the law'. In other words, proper and suitable behaviour within a society. In this sense lagom is a social construct that relates to creating a just society, encouraging members of society to abide not just by the written law, but also to practice common sense.

Today that original meaning has modernised and shifted slightly, but it still carries the same

social impact. Lagom is often translated into English as 'not too much, not too little, just right'. It's a word that implies understanding what the extremes are and finding the moderate path in between the two. It means behaving appropriately, eating the right amount, and on the flip side, celebrating the right amount too. In Swedish it's an all-encompassing word, one that can be directly applied to almost every element of society. Lagom food, lagom drink, lagom work hours; the idea that a balanced amount of everything leads to a just and equal society and, overall, a good life. (Document analysis: Live Lagom, Balancing living the Swedish way, Brones 2017)

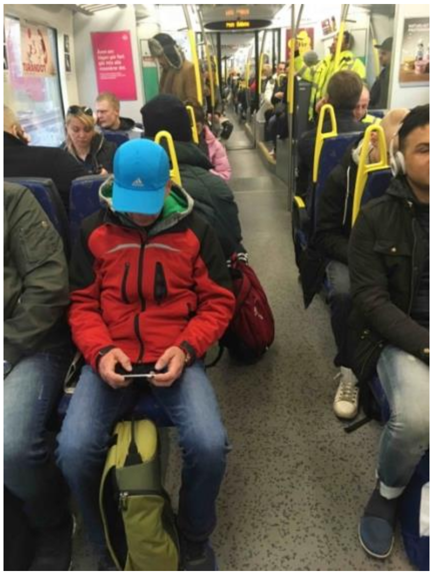

Figure 3. Train at 6.07am

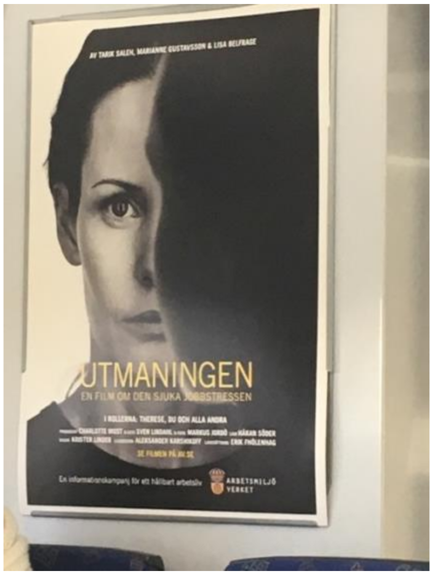

Figure 2. advert from the 'Work environment government agency'

Lagom work hours would be nice; I step onto the 06:07 train and I can't find a seat. This morning I have a 07:00 meeting with AIK's head coach and his staff at Karlberg… I customarily check my phone and see an email from Saturday night, sent at 23.28 (by an AIK colleague). "We don't work full time, we work all the time" is the motto fondly (sometimes exasperatedly) attributed to the youth club's sporting director, who has been active in the club for 54 years. I wonder at the wisdom of such a slogan in today's world… another friend was diagnosed with psychological burn out and discharged from work on sick leave over the weekend…To enforce the issue my eye catches an advert from the 'Work environment government agency' (Arbetsmiljöverket) promoting a film called 'The Challenge: A film about the sick job stress'.

1.2 Privatisation, folkhemmet and complex problems

As the train slides into Älvsjö station a McDonalds advert commands my attention, ‘En lite lagom stor’, (see Figure 17) it roughly translates to ‘Just about big enough’ double EL MACO 28 Sek. Two thoughts emerge. First, that is amazingly cheap (for Stockholm) and second, I wonder if people care about big business claiming lagom? Leaving the station, I’m accosted by another advert (see Figure 15), this one is inside the train and asks: *Do you want your carrier to go on rails? Search for Thoren Business school.* Gymnasiums (senior high schools) are unashamedly advertising for students and according to media reports, some schools have started to market courses to prospective ‘YouTubers’ and ‘Influencers’ (monetised social media entrepreneurs, such as bloggers). Are schools adapting to a changing media landscape? Or planning to profit from the latest market trends?

At his sleek apartment in the hipster mecca of Telephonplan, Andreas explained the extent of school privatisation to me last weekend; “in the 1990’s they started privatising the schools… today 40% of schools are private” (Field note: informal conversation 26th March).

I was even dragged into the prospective student market, holding a training session to attract pupils to Norrviken school (via their football program in association with AIK). Government

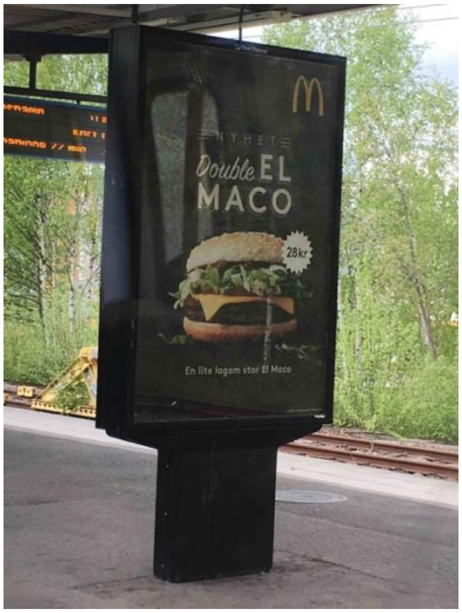

Figure 6. El Maco advert

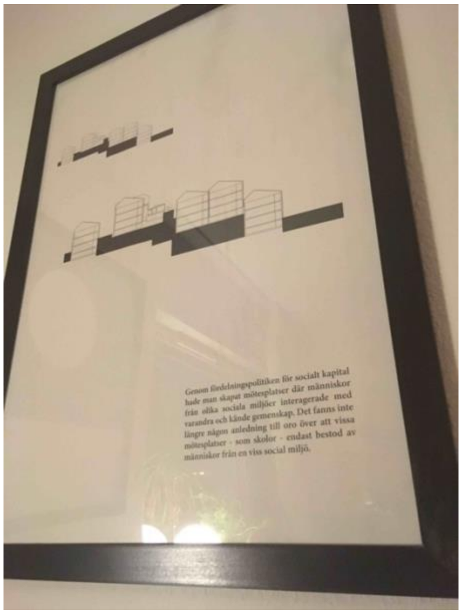

Figure 7. A framed picture commemorating the former role of school

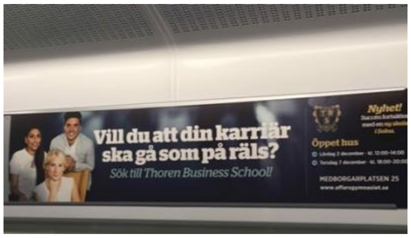

Figure 4. Thoren Business school advert

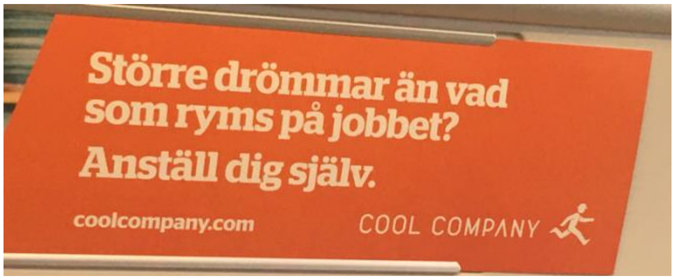

Figure 5. Coolcompany.com advert

funding is received for every student enrolled at a school and privately-owned schools intend to make a profit, however, historically Swedish educational institutions have embodied other ideals. Above Andreas' sofa, amid the fashionably designed interior, I remember a framed picture commemorating the former role of school (see Figure 18):

> 'Through the policies of distribution of social capital one had created meeting places where people from different social backgrounds interacted with each other and felt a sense of belonging. There were no reasons anymore to worry that some meeting places, like schools, only consisted of one category of social background' (Field note: Artefact collected 26th March).

As the train speeds along, I brood on the incessant advertising, I pull another advert down – this one, for coolcompany.com (Figure 16) was positioned in front of a window obscuring the precious winter daylight – and as the world flies by the smoke pouring out of dilapidated caravans catches my eye. I've been told that Stockholm's beggars live beside the railway tracks in these old, cold, caravans. My mind drifts back to a recent conversation:

> Alexi: The thing with the beggars in Stockholm is that it's a really difficult situation to handle and know what to do, because a lot of them come from European Union countries, making it really difficult for the government to know how we should step in? How should we handle this? Because they are by definition… allowed to be here, of course.
>
> James: But they're not part of the Swedish social system?
>
> Alexi: They're not part of the system and they're not applying to be (part of the social system) so it's difficult to know. I feel like we are in a situation we've never been in before, the government doesn't know how to handle it, Swedes don't know how to handle it…
>
> Lilly: We are used to the government stepping in, to sort things out, but it doesn't apply in this case, does it?
>
> James: Is it organised in some way?
>
> Lilly: DN (national newspaper) did a good article on it, and it depends what you mean by organised. They aren't exactly coming one by one, but they couldn't find anything suggesting that it's a big organised plot to make money.
>
> Andreas: It's not a franchise…
>
> Sam: But isn't that back up for debate again now? They found something.
>
> Andreas: Yea something like that people are selling places on the street? Or something like

that.

Dave: I think the one (journalist) that made the investigation in the newspaper has changed his mind and he does think there are elements of organisation.

Alexi: I think it's something that's going to grow.

Dave: Yes, I think it evolved over time.

Andreas: Yea because organised crime is always going to use those who are weak (vulnerable) in society, that is the easiest target to get money.

(Field note: Informal conversation, 30th April 2017, 12.42 pm)

As the train flies past the ramshackle caravans, I think about the new HSB apartment building that I walked out of this morning. Perhaps it's a modern interpretation of the traditional Swedish ideal of Folkhemmet explained to me by Alexi.

I was in an exhibition a year or two years ago at Nordiska Museet that was all about folkhemmet, meaning the people's home, folkhemmet being that we should view society like we are all one kind of family and we should all help each other out. Because (back) then they were building a lot of new apartments moving a lot of people from places where they didn't have a bathroom, didn't have a toilet, one room, to these standardised apartments. So, these were standardised apartments that the government built whereby everyone looked the same, it was clean it was efficient. You could see an engineer has designed it, it was about getting as big an apartment as possible, for as many people as possible in a small space…and they were going to look the same and everyone had it the same. (Field note: informal conversation 30th April, 12:36 pm)

Figure 8. Pictures from the Folkhemmet exhibition at the Nordiska Museet (Nordic museam)

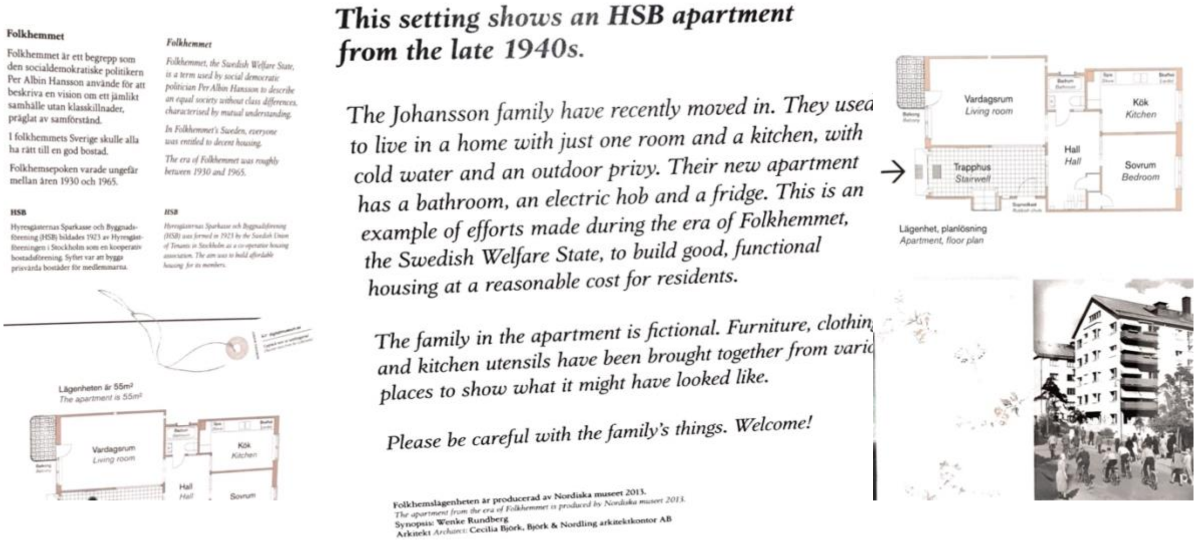

I recall visiting the Nordiska Museet (Nordic Museum) and seeing the same exhibition, an installation commemorating folkhemmet and Sweden's success as a welfare state. As the train efficiently flies into the city, it passes a few more derelict locations dotted with caravans.

> Every time I see the random placement and shabby exteriors of the caravans, I can't help noticing how they stand in stark contrast to the backdrop of ordered exteriors and fanatically 'designer tidy' interiors of Stockholm's suburban apartment blocks. Walking into Pontus's apartment for the first time and getting the guided tour – the apartment tour is a Swedish institution – he knowingly exclaimed; "Welcome to the most boxed in country there is… everything has its place" (Field note: 12th April 2017).

An obsession with order brings to mind a conversation with Stephen after last week's parent presentation at the football club.

> Stephen: Then they ask (Parents) "yes but we're in Sweden, so what influences our style of play?" So I said I don't research this area but for me I think that… well we come from a Newtonian paradigm which in itself is very linear, then we have built all our welfare through our industry, the only industry in Europe that hadn't collapsed after World War II. So we had a lot of positive encouragement from going to the factory lines, standing there, working hard and producing, and I think we relate success to planning and linear models in general… but maybe since football is dynamic and unpredictable maybe we need to question that, because of the nature of the game, not because we want to be someone else but because of the nature

of what football is…."

(Field note: Informal conversation, 5th May 2017)

Stephen was recently headhunted by AIK to become their head coach (on the boy's side of the football club) and head of education throughout the football club. He'd previously worked for a smaller Stockholm football club and was an influential part of the working group designed to re-evaluate AIK's academy. Stephen does not come across as a stereotypical Swede, but he has lived in Stockholm for his whole life.

1.3 Engineering success & Norse Mythology

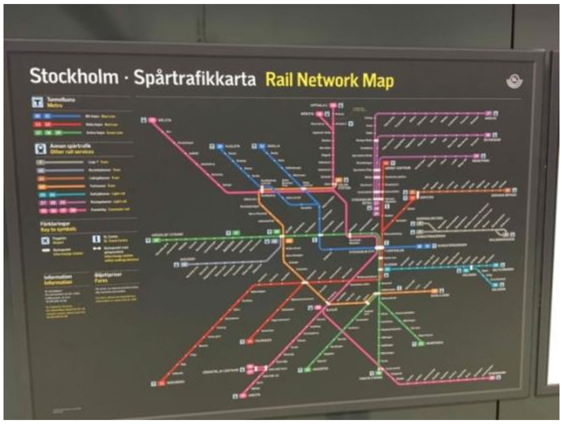

Figure 9. Stockholm Rail Network Map

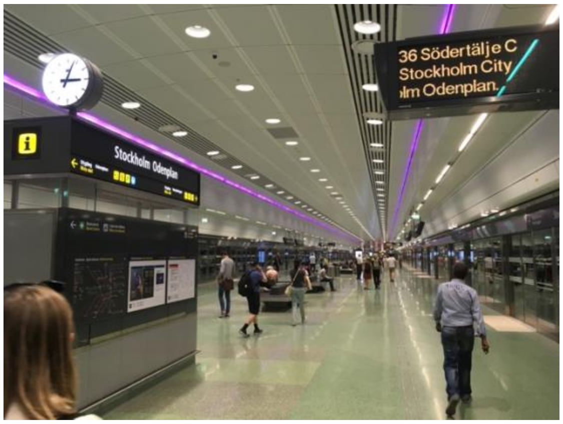

Figure 10. Underground at Odenplan station

Today, the pendeltåg (commuter train) glides along, embodying the engineering success Stephen highlighted. Organised linearity and mechanised order mean that I cover the 13km into the city centre in 22 minutes making 5 stops before arriving at the newly opened station under Odenplan; a central city square named after the Nordic God, Oden.

Odenplan station is one of two new subterranean stops built as part of a 7.4km railway tunnel running under Stockholm. But rather than looking subterranean the Odenplan platform has lighting which shifts, mimicking the sun as it fades in and out of clouds. Adding to Stockholm's already impressive public infrastructure and aiming to ease congestion, the new railway link is a feat of engineering that took ten years to complete. The station's reference to Norse mythology reminds me of a popular newspaper article (about a research project) that highlighted some perceptions of Sweden and Norse Mythology. The article came out on Torsdag (Thursday) or Thors-day (named after the god of thunder) and described the research of Patrik Hermansson, who went undercover to research right wing extremists, the article read:

> The Head of Research suggested infiltrating London Forum, a convention that attracts right-wing extremists of varying piousness, from the ethno-socialists in the British National Party to downright Holocaust deniers. Hermansson quickly connected with the founder of the forum, Stead Steadman, who as many others in the subculture nurtures a fetish for Sweden.
>
> He learned Swedish and Icelandic by studying the Edda. He worships Thor and Oden. He had visited a right-wing extremist forum in Stockholm and was surprised by how presentable the right-wing extremism looked in Sweden. Educated people with proper jobs. I made myself look like one of those people. Then I became interesting to him. I basically became his Swedish teacher. We spent hours together."
>
> Patrik Hermansson pretended to be upset by the development in Sweden.

> "I presented myself as a political refugee from the left-wing dictatorship of Sweden. I talked about no-go-zones. That Sweden was collapsing under mass immigration. Many fascists worship Viking-Sweden, the idea of a clean white race and a pure, original masculinity. Something that the so-called cultural Marxism and immigration has belittled and ruined. The idea of a lost paradise."
>
> (Field note: Dagens Nyheter 20-9-2017)

The Norse mythology I've heard tells of a diverse and fallible pantheon of Gods and paints a picture very different to that of 'pure original masculinity'. I recall one popular story whereby a cross-dressing Thor is the transgender hero. After Loki's somewhat malevolent mischief, Thor must pretend to be the goddess Freja and marry a Giant to reclaim his fabled hammer, Mjölnir (Document analysis: Norse Mythology Neil Gaiman, 2017). Perhaps white supremacists cherry pick their Norse mythology?

Thinking about white supremacists reminds me of Sweden's national day. As we walked into a beautifully green, wooded area (see Figure 22) along one of Stockholm's many shorelines Stephen explained; "These parks were where the neo-Nazis used to hang out, about 10 to 15 years ago but now the people have claimed them back". With thousands present, the stage was constantly obscured by an orgy of dancing national flags (Figure 23) and during the musical interludes –when the fusion of reggae, dance and electronic music briefly stopped– someone would jump on the microphone and yell: "Stockholm needs more openness and tolerance... hands in the air for a more tolerant and loving Sweden." (Field note: informal conversation 6th June 2017).

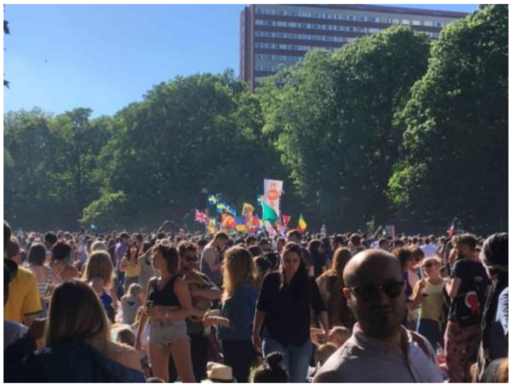

Figure 12. Dancing national flags

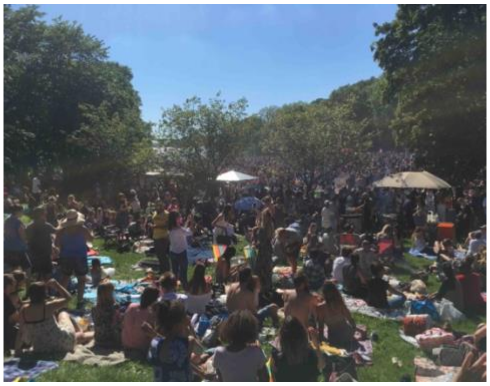

Figure 11. National day celebrations

1.4 Equality and diversity

The memory morphs as I step off the Odenplan station platform and onto the rainbow escalators leading up and away from the subterranean platform (see Figure 24). Mesmerised by the spectrum of colours I recall the captains' armbands worn by the organisers (Figure 25) at Stockholm sports federation's conference (Stockholms Idrottsförbund) and Stephens' comment that "all the (football) clubs in the Swedish first division will wear them this season" (Field note: informal conversation, 25th of March 2017). The conference made reference to people's fundamental right to participate in sports and the UN convention on the rights of the child. Stephen explained that "the federations are really pushing a child-centred approach, this is a big event for Stockholm sport" (Field note: Informal conversation, 25th of March 2017). When discussing the diverse differences of world-class footballers like 'Zlatan eller (or) Messi' the speakers highlighted that 'everyone is equally different'. A message echoed on the Swedish Football Federation's website saying; "Everyone is different, different is good" (Alla är Olika, Olika är Bra, see Figure 26). The call for sporting structures to move from elitist selection pyramids to inclusive participatory rectangles was reinforced by the call to include as many as possible, for as long as possible, as well as possible, in the best environment possible. While the rhetoric is undoubtedly positive, sport policy has struggled to drive real change. A professor at GIH (Swedish school of sport) explained the timeline of slow progress like this; 'I'm tired after twenty years' (Field note: Informal conversation, 7th February 2018).

However, in June 2017 AIK youth football released a statement (translated into English) echoing these ideals and announcing a significant change in its academy structure:

"The debate around a healthy childhood and youth sport has been going on for some time and

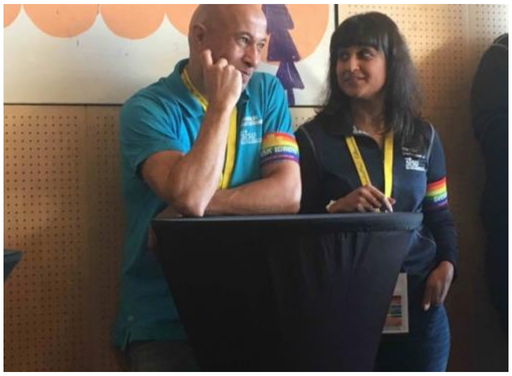

Figure 15. Rainbow Captains armbands

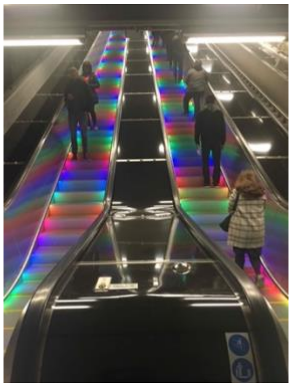

Figure 13. Rainbow escalators in the Metro

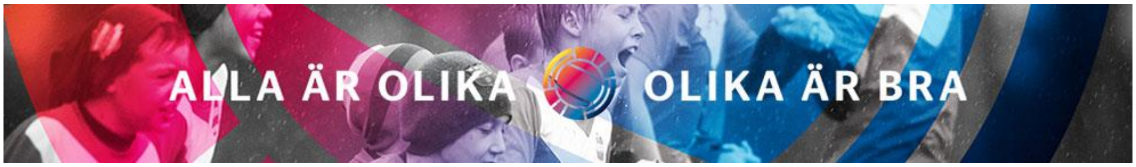

Figure 14. Swedish Football Federations website; "Everyone is different, different is good"

continues to engage many people, with different backgrounds and objectives. Criticism

(aimed at football clubs with academies) is often grounded in the children's rights perspective with reference to the UN Convention on the Rights of the Child, the documents by the National Governing Body for Sports (riksidrottsförbundet) and the Swedish FA's (SvFF) book 'Spela. Lek och Lär' (Play the game, Play and Learn).

Based on this background, AIK appointed a working group as well as a reference group (including technical directors in ice hockey and floorball, a Bayern Munich scout, researchers in child-youth sport and local politicians) to review the activities of children from eight to twelve years of age and the consequences it has for the rest of the club."

The club's definitive goals, and specifically with this project are as follows:

1. We want all children in AIK to feel good.

2. Increase the development and promotion of players to our own senior teams, as well as increase the number of players in the U16-U19 and F16-F19.

3. We also want to follow relevant control documents in Swedish child-youth sport. In other words, encouraging children and young people in AIK to develop an interest in sports in general and in particular, to keep them playing football in AIK for as long as possible and to continue playing sport as much as possible through life.

Based on this review (conducted in 2016 and early 2017) a decision was made for a change of focus on the activities in child-youth football for 8-12 year-olds in AIK. The club will delay its academy selection until the age of 13. There will be no selection process in this age group. Instead training groups will be formed with increased support from AIK, through a deliberate investment in resources to support the coaches working within this age group.

(Field note: 9th June, Swedish English translation by Mark O'Sullivan).

I remember discussing AIK's organisational changes over 'fika' – the Swedish institution of coffee breaks and small cake-ish treats – with AIK's younger academy coaches:

Pontus: "Could be the first step in a much bigger change"

James: "Do you think other clubs…."?

Pontus: "No I think the big change will be within AIK"

Carlos: "Hammarby and BP will not follow, they will go the other way and start selecting them at 6 years old"

Pontus: “And then they’ll be at the birth of children, testing them… and then they’ll be figuring out how to spot the woman who’s going to give birth to the most talented player”.

1.5 Part 1 summary

In the first section subtle tensions (contradictions) are introduced, specifically people’s desire to simultaneously “fit in” and “stand out” in social situations. More broadly however this section introduces the theme of lagom and contrasts the traditional desire for a balanced, healthy and “lagom” life against the modern reality of extreme work hours.

The next section Privatisation, Folkhemmet and Complex problems highlights the tension between old and new ideals. More broadly, these contrasts illuminate a tension between (neoliberal) competition and (social) collaboration: a Meta theme that runs throughout the narrative. Conversations about Stockholm’s beggars acknowledge the world’s complexity and contrasts are drawn between the secure, ordered apartment blocks of “Stockholmers” with the randomly placed caravans of the city’s beggars. Here a deeper question is posed; is the historical success of linear planning stifling approaches that are better able to deal with complex (wicked) problems?

Engineering success & Norse Mythology follows on from the previous section and introduces Odenplan station, one of the city’s newest artefacts which exemplifies engineering success for the collective good. The narrative moves on to introduce documentary analysis of extreme right-wing ideals and contrasts them with field notes (conversations, artefacts and espoused values) taken on Sweden’s national day. In this section of the narrative, extreme forms of elitism and exclusion are contrasted with vividly espoused values of openness, tolerance, equality and diversity.

As the narrative moves briefly into the realm of sport it highlights the call to “include as many as possible, for as long as possible, in the best environment possible”, an ideal reminiscent of folkhemmet’s collective intention of providing “as big an apartment as possible, for as many people as possible, in a small space”. While few sports’ organisations take up the more inclusive call, AIK have changed their organisational structures to better align with it. However, as this section draws to a close, conversations suggest that few football clubs will follow the lead of AIK, with the trend to select younger and younger players into the traditional, elitist, talent development pyramid continuing.

2 Part 2

2.1 The museum of America

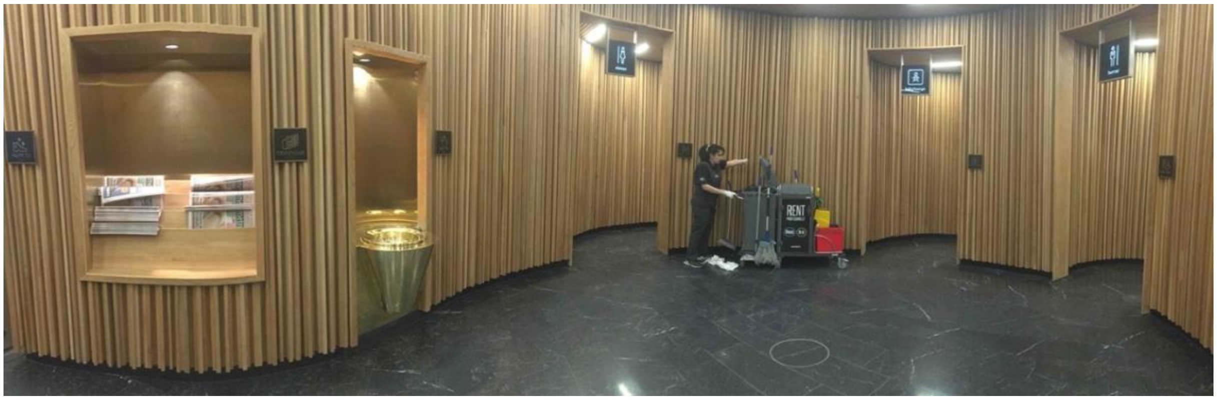

Figure 16. Golden fountain at mall of Scandinavian (museum of America) toilets

I step off another train at Solna Station and walk towards the AIK offices; it's snowing so I'm forced through the Mall of Scandinavia, or as a visiting friend called it "the museum of America". With that said, there is no escaping the Scandinavian-ness of this mall, or perhaps it's the Stockholm-ness? From the sleek wooden bench at the entrance to a gold leaf drinking fountain and newspaper resplendent public toilets that look like 'champagne consumerism'. The 'mysigt' (cosy) Nordic lighting and the general spaciousness demonstrate a Scandinavian sensitivity to design and temper the obtrusive almost garish advertising of most malls. However, there is no escaping the manic use of one word, which is obsessively repeated on store windows:

"Fashion!!! Fashion!!! Fashion!! Fashion!!! Fashion!!! (Field note: 19th March 2017).

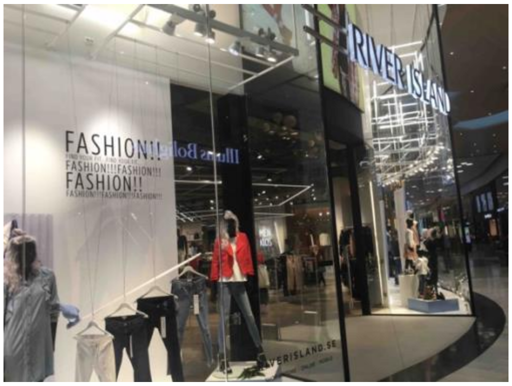

Figure 17. Fashion, Fashion, Fashion

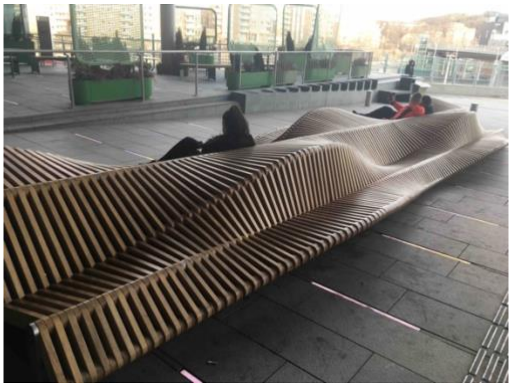

Figure 18. Innovative Scandinavian design bench

I cringe and recall an article (in Svenska Dagbladet; a national newspaper, see Figure 30) detailing the international recognition of Stockholm's collective obsession with fashion and design.

> With an inner city that makes up the world's most anxious square kilometres, Stockholm is consequently one of the most popular test markets for several major companies in technology,

fashion and design. Both in domestic and international newspapers, it is clear that Stockholm is "trend-sensitive" when it should instead be "trendy"…

The sociocultural-historic role of fashion and design were explored in extensive exhibitions when I visited Stockholm's Nordic Museum:

> The dress code is, and has been, one of the most obvious markers of social status and gender. The costume denotes connectedness within the category of people that wears it, but at the same time it marks the exclusion of other groups.
>
> (Field note: Iklädd Identitet, Nordiska Museet, 11th September 2017).
>
> In the Nordic region, the period after World War II is seen as the golden age of design… People had more money, which meant they could consume more… Design became a way of expressing yourself, not just for individuals but also for nations. (Field note: Nordiska Museet, 11th September 2017).

Expressing individuality and demonstrating exclusivity seem to be neoliberal themes that run beyond fashion and design today:

> The city of Stockholm markets itself as the good city; it is the rich commercial city with its 'vibrant urban life where everything costs and all folklore is based on the fact that money is constantly circulating' but as the writer Alexander Berthelsen expresses it:

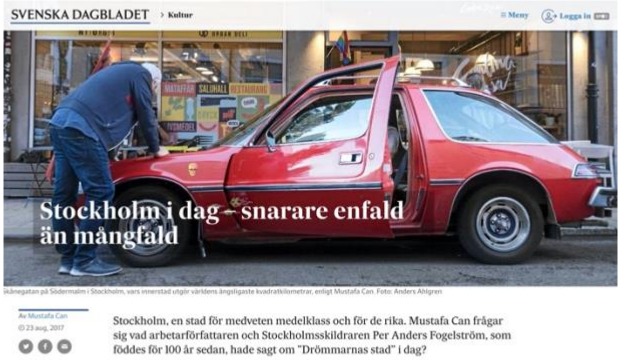
SVENSKA DAGBLADET › Kultur
≡ Meny Logga in
Stockholm i dag – snarare enfald än mångfald
Skånegatan på Södermalm i Stockholm, vars innerstad utgör världens ängsligaste kvadratkilometrar, enligt Mustafa Can. Foto: Anders Ahlgren
Av Mustafa Can
23 aug. 2017
Stockholm, en stad för medveten medelklass och för de rika. Mustafa Can frågar sig vad arbetarförfattaren och Stockholmsskildraren Per Anders Fogelström, som föddes för 100 år sedan, hade sagt om "Drömmarnas stad" i dag?

Figure 19. Svenska Dagbladet: Stockholm creates more anxiety than dreams

> 'Stockholm is Sweden's most beautiful city, but also the most segregated place, ethnically and economically and spatially, with clear boundaries between the inner city's districts and the suburbs'… What would Per Anders Fogelström (the working-class writer who described how the people changed society in a collective effort), have said about the City of Dreams today? (Fogelström was born 100 years ago and wrote a book about Stockholm called 'The City of Dreams').
>
> What about the effects of public services being sold and the poorest and richest groups becoming increasingly geographically isolated? …

(Document analysis: Svenska Dagbladet, 21st August 2017, translated from Swedish).

2.2 Design: Exclusive or inclusive?

Alongside public services, the rental properties of the Folkhemmet era are also being sold. A current age of privatisation means cash (economic capital) or contacts (often determined by social capital) determine where you live. As the inclusive ideology of Folkhemmet continues to fade the waiting lists for rental apartments continue to grow; most range from 10-20 years. Eighty years ago, Sweden dealt with increasing urbanisation differently, an approach celebrated at the Nordic museum.

> During the 1930's, Finland and Sweden were the countries with the most densely populated cities in the world. The housing problem became an important political issue and the idea of Folkhemmet (the Swedish Welfare State) was coined by Sweden's Prime Minister Per Albin Hansson.
>
> There was no room in the apartments for large, awkward furniture. Homes should be functional and easy to look after. Artists were involved in industry, which gave rise to industrial production with an artistic expression. Standardised production resulted in cheaper products more people could afford. (Field note: Nordisk Muset, 11th September 2017).

The idea that art and industry should integrate to maximise space and create affordable (inclusive) solutions may have helped foster Sweden's most recognisable global institution: IKEA has thrived by providing people the opportunity to:

> James: Do so much with such small spaces, living in big cities it seems… that a lot of the creativity comes from the constraint of a small home?
>
> Alexi: I understand what you mean, IKEA makes a lot of good storage solutions where the bed can go into the wall and tables can fold together to make something else, and IKEA has built a whole concept around this, and IKEA is a big part of Swedish culture."(Field note: informal conversation, April 30th)

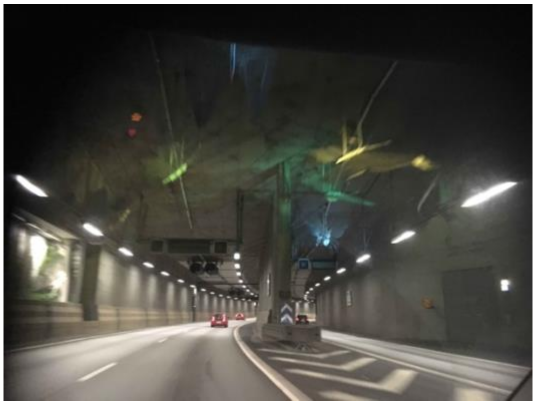

Figure 20. Artwork in the tunnels under Stockholm

Contrary to the individualism, elitism and geographic isolation somewhat evident in Stockholm today, the golden age of design may have emerged from integration and inclusion:

> In the Nordic region, the period after World War II is seen as the golden age of design. The

> borders, closed during the war, were opened again and the influences from other countries and cultures were able to come in. (Field note: Nordiska Museet, 11th September 2017).

Today the aesthetics of art and the efficiency of engineering merge throughout the city. The effect maximises space and demonstrates a sensitivity to light, another key constraint in Nordic countries. I remember that Stockholm's metro is celebrated as the world's longest art exhibition and the roads burrowing their way under the city are expansive and intricate, yet spacious and dotted with luminous artwork (see Figure 31).

2.3 Exclusivity bleeding into anxiety

As I continue to walk the Mall of Scandinavia, it is hard to not become consumed by the neoliberal rhetoric of economic elitism, despite the histories of collaboration and policies of inclusion. While this city contains many symbols that espouse equality and celebrate diversity, the consumption of fashion, property and design often feel like subtle tools for social comparisons, divisions, evaluations, judgement and insecurity. A dinner conversation with Stephanie (Stephanie is approaching retirement and has lived in Stockholm for her whole life, teaching young children and now teaching the teachers) highlights the subtle dance between the collective ethos of folkhemmet and the neo-liberal narrative of consumption and social competition.

> Stephanie: It might be a paradox that in general we think no one should be above anyone else, but then each individual thinks that they would like to be like that (a little bit above others), but maybe in every group in a very quick moment you position yourself in a hierarchy and you very quickly see who is below you and who is above you. And in a general way this is not ok. So maybe this increases the wishes to… hide that you are doing that. It's not socially accepted but it's sneaked in and maybe it makes us more aware of the subtle nuances. So, we distinguish ourselves in very subtle ways.
>
> (Field note; informal conversation, 6th April 2017)

Stephanie's explanation initially exemplifies the typically Swedish/Norwegian value 'jantelagen' – the ideal that no one is above, or better than anyone else – but moves on to problematise its expression and the emergence of subtle comparative nuances. Writing about the frustration of living on what he describes as the socially sanitised island of Södermalm, Mustafa Can gives insight into the subtle nuances of elaborate coolness and its side effects:

> Now I'm suffering from an increasingly paralysing anxiety, and wonder what the hell I am doing in this homogeneous enclave that would better serve as a social anthropological theme park for elaborate coolness and hypocritical spontaneity – a place where people take selfies with refugees (at risk of being deported) to increase their social capital… (Document analysis: Svenska Dagbladet 21st August 2017, translated from Swedish).

When you first arrive in Stockholm, or Södermalm, or even this mall it is easy to be swept away by a polished façade. It's like arriving at a Scandinavian airport; clean, tidy, aesthetically pleasing, cleverly designed, spacious and well lit. And while it is easy to fall in love with this polished aesthetic, it's equally easy to be swallowed up by the neoliberal cocktail of working hours, social expectations and emergent anxieties; popularly referred to by a generation of Stockholm parents as Livspusslet (the Life Puzzle);

> Lilly: For people of our generation the attempt to navigate and balance our lives is overwhelming and it's being called 'the life puzzle'. Which captures the spirit of trying to be healthy and feed your kids organic food, whilst also working out and moving on in your career and having a happy marriage. But it's also become a buzz word; it's become a thing in itself to refer to it, because it's a sign of 'well I'm something and I'm onto something because it's challenging…'
>
> James: Like a badge of honour? It's going 'I'm really busy and important'?
>
> Lilly: Yea and I'm also very aware of how important it is to fit all these pieces together… the word is troublesome because it suggests that there is a solution and a way to make all the pieces fit…and that you can balance it all… the connotation it brings is a never ending constant struggle and rush… and like baking a cake for the meeting, while not having the kids sitting in front of the iPhones too long, while cooking a nutritious organic meal and having perfect sex with your partner…
>
> (Field note: informal conversation, 15th September 2017)

I remember when the life puzzle and having 'mycket att göra' started to become overwhelming:

> I don't know why, but I am feeling so frustrated with life, and this frustration is starting to boil over, perhaps the situation at the bank was an example.
>
> I feel exhausted just thinking about it… I'm working too much, this seems to be a systemic problem in Stockholm, or is it worldwide? Edvin has a saying that is re-told throughout AIK, 'we don't work full-time, we work all the time'. And I'm trying to immerse myself in this club; collecting data as well as fulfilling other expectations.
>
> I remember one of the first Swedish expressions I was taught 'mycket att göra'. Ben told me to say this when I went to the AIK's offices because 'that's what they all say, all the time'. Roughly it translates to 'much to do' or 'very busy'… or perhaps 'very stressed'…

> When I got home tonight, I was exhausted, and I kept thinking is this it? Is that all there is to Stockholm life? Bullshit busyness? (Reflexive journal: September 1st, 2017).

"We are basically cucumbers with anxiety" (Field note: Informal conversation, 26th August 2017) is how a friend describes the human condition in Stockholm: 70% water, 30% anxiety. I was starting to get a taste of this…

2.4 Progressive but neoliberal?

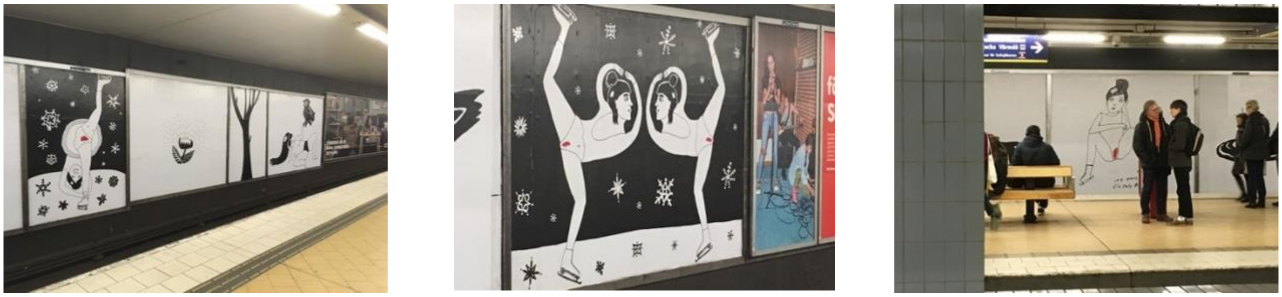

Figure 21. Feminist artwork in the Metro

There is little doubt that Sweden is very progressive, both socially and politically. Compared to most countries the whole political scale can be conceptualised as 'shifted to the left', making Sweden's more conservative right-wing policies comparable to the centre on other political scales. This socially 'aware' population is seen as fertile ground for 'people's movements' like the #metoo campaign. Dagens Nyheter (a national newspaper known as DN) explains:

> In the world's most liberated country (Sweden)… tens of thousands of women chose to say, "me too." The actors were first out. Then followed the singers, lawyers, women in the technology industry, teachers, politicians, academics, athletes, journalists, cooks, doctors ... So far, 70,000 women in some fifty sectors have signed the protests. (Document analysis, Dagens Nyheter, 4th December 2017)

Even compared to its Scandinavian neighbours, Sweden, and in particular Stockholm, is viewed as progressive. Moving back to Stockholm from Copenhagen a coach told me that the prevalence of sexist remarks makes "Copenhagen feel like it is ten years behind" (Field note: 28th February 2018). While in Stockholm the feminist ethos is evident in many cultural domains ranging from politics to art (see Figure 32).

I start to consider the cocktail of progressive politics, high performance and subtle forms of differentiation (like the consumption of fashion). Does this cocktail create more social expectations; more social rules to follow; more faux pas to make; more opportunity for judgement and insecurity; more pieces to life's puzzle? Rather than high society, does it create high anxiety?

In the mall I walk past H&M and see their ‘conscious’ clothing collection, next comes Nike’s semi-fraudulent attempt at environmentally friendly T-shirts and finally I walk past ÅHLÉNS. This reminds me of another collection of T-shirts, stickers, cards and clothing at Stockholm’s Art College Christmas fair: This collection all had one word on the front, copying the branding of Sweden’s most iconic, department store ÅHLÉNS it said ÅNGEST (anxiety). If fashion is supposed to say something about who we are, perhaps this is more appropriate than anything else. As big corporate clothing brands seem to realise, many Stockholmers want to portray an environmentally friendly persona, but as the artists recognise, an emphasis on fashion may be fostering anxiety.

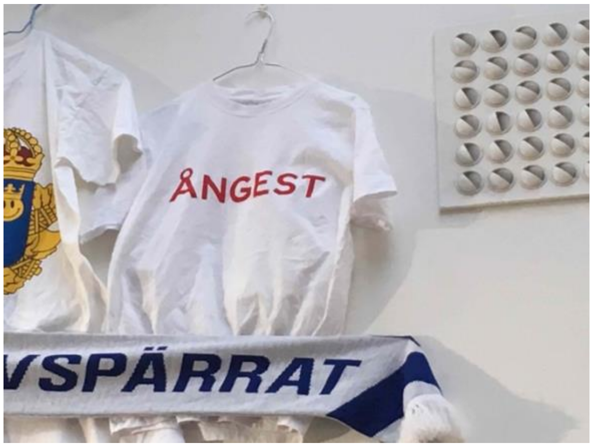

Figure 22. Mocking the Swedish department store ÅHLÉNS by renaming it ÅNGEST (anxiety).

While the quantum of social expectations may lead to anxieties I wonder if tensions between ideologies exert pressures too. I remember writing:

> We walk up rainbow coloured escalators espousing equality while being bombarded with the rhetoric of individualism: Advertising suggesting that you, the individual, should be striving to stand out, get ahead and be on top! I wonder what happens when the neo-liberal narrative of “individuals get ahead by working hard” and Sweden’s historical ideal that “everyone is equal, and we look after the collective” collide? How do people deal with this hypocrisy? Anxiety? Tension? Frustration? Anger?
>
> (Reflexive Journal: May 20th, 2017).

When I stop to think about it, I have never encountered so many people on sick leave with psychological burnout as I have in my time in Stockholm. I recall a newspaper article saved on my phone… I skim down to a particularly relevant section:

> What greater indictment of a system could there be than an epidemic of mental illness? Yet plagues of anxiety, stress, depression, social phobia, eating disorders, self-harm and loneliness now strike people down all over the world. The latest, catastrophic figures for children’s mental health in England reflect a global crisis.
>
> There are plenty of secondary reasons for this distress, but it seems to me that the underlying cause is the same everywhere: human beings, the ultra-social mammals, whose brains are wired to respond to other people, are being peeled apart. Economic and technological change play a major role, but so does ideology. Though our wellbeing is inextricably linked to the lives of others, everywhere we are told that we will prosper through competitive self-interest

and extreme individualism."

(Document analysis: Telegraph article: Neoliberalism is creating loneliness, that's what's wrenching society apart 12th October 2016.)

My phone beeps and a notification pops up, I click the YouTube link and Sweden's Prime Minister Stefan Löfven is standing alongside President Donald Trump at a White House press conference. Löfven is espousing the countries shared values:

"Sweden and the United States are two of the most innovative economies in the world…Swedish prosperity is based on collaboration, competition and free trade." (Field note: White House press conference, 6th March 2018).

While that is nice in theory, the routine patterns of life suggest that collaboration is consistently 'trumped' by the aforementioned 'competitive self-interest and extreme individualism' of neoliberal free market beliefs. However, in certain situations this overtly competitive emphasis melts away. Recalling the terror attack of April 2017 Stephen noted:

"The values of open-minded caring people shone through when Stockholm was attacked. However, the fact that such an act is required for these values to emerge shows that while these values are there… they are buried deep under the neo-liberal crust of modern western society."

(Field note: informal conversation 13th March 2018)

These open-minded, socially orientated values manifest in the social media hash tag #openstockholm. As public transport ground to a halt and thousands of people (myself included) were stranded in the city, people on social media responded by offering food and shelter.

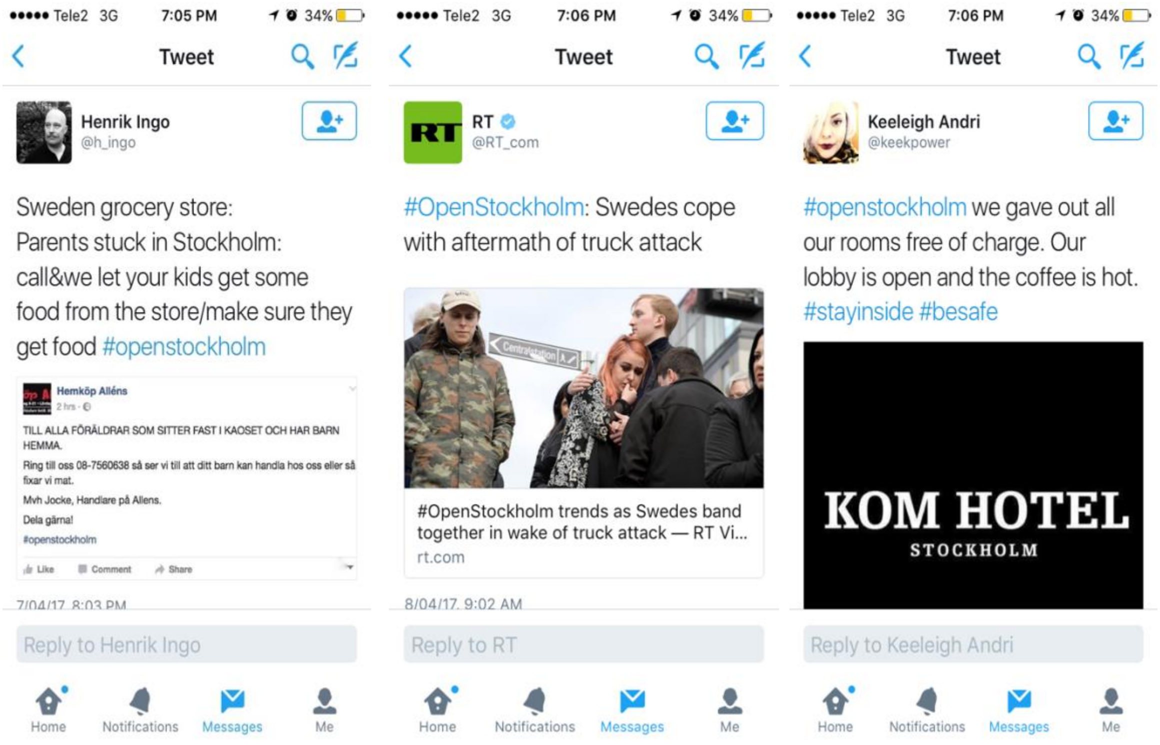

Figure 23. Support is offered on social media (Twitter) using the #openstockholm hashtag

However, once normal life is resumed, neoliberalism continues to inspire a growing inequality, according to a popular newspaper article by journalist Mustafa Can some facts are worth noting:

> The difference between the expected life expectancy of a low-education area in Vårby and a high school in Danderyd is 18 years.
>
> Between 1990 and 2015, average income for residents in the inner city increased by 64 per cent, while the increase in Skärholmen was only 1 per cent. During the same period, the highest income group had increased its share of the population in the inner city by 62 percent.
>
> In Rinkeby, 42 percent of families live in relative poverty. In Södra Ängby only 2.
>
> (Document analysis: Svenska Dagbladet 21st August 2017, translated from Swedish).

The current reality of economically inspired geographic isolation is captured by the graffiti scrawled alongside a walkway marking the boundary between a "million program housing project" and an affluent neighbourhood in Stockholm: "It's easier to see the end of the world than it is to see the end of capitalism" (Field note: 5th September 2017). My mind drifts back to a conversation with Andreas at a friend's wedding, he said:

> "We live in a mono-polarised world, we used to have USA vs. Russia. Socialism vs. Capitalism but today we just have Capitalism, which really only emphasises one set of values" (Field note: Informal conversation May 20th, 2017).

Perhaps Sweden's geographically middle position between east and west (lagom) has helped balance the competition of capitalism with the collaboration of socialism? Maybe this geographical position has shaped an ability to see both points of view and mediate international disputes, finding a middle path? Is this another emergent feature of a value in lagom: not too much, not too little, just the right amount? Either way I'm left wondering if Stockholm life is really characterised as lagom? Or is one set of values tipping people towards a competitive extreme?

2.5 Part 2 summary

Part 2 follows the narrator through the Mall of Scandinavia (nicknamed the museum of America) and begins by emphasising the Scandinavian subtleties of an artefact that globally represents American corporate capitalism. As private consumption reigns, documentary analysis describes how the exclusivity evident in the consumption of fashion is also evident in the consumption of public assets throughout the city, leading to a growing segregation.

The next section, "Design: Exclusive or inclusive?" utilises field notes from the Nordic Museum to contrast the historical emergence of Scandinavian design, with the current exclusivity of fashion. Creative collaborations during the collaborative era of folkhemmet may have sown the seeds for the standardised production and affordable design we see today. IKEA is the iconic example. The next section of the narrative "Exclusivity bleeding into anxiety" introduces some emergent features of the macro tensions between histories of inclusivity and collaboration, versus the current realities of competition, elitism and economically driven exclusivity. Leading to the description of Stockholmers as "basically cucumbers with anxiety, 70% water, 30% anxiety."

The section "Progressive but neo-liberal?" begins by considering the political landscape of a country described (by a national Swedish newspaper) as 'the world's most liberal'. The question is raised, as to whether a mix of high performance, perfectionism and progressive political correctness induces anxiety? Artefacts recognising Ångest/anxiety are introduced alongside personal reflections that highlight conflicting feelings. In day-to-day interactions however, collaboration (a genesis of creativity) is regularly marginalised by the dominance of competition and extreme individualism. While exceptions are introduced, like #openstockholm, open-minded, socially oriented values are seen to reside under the "neo-liberal crust of modern western society". This section concludes with some emergent examples of Lagom and questions the current balance of life in Stockholm.

3 Part 3

3.1 Bosman & Business

I exit the Mall of Scandinavia and end up sandwiched between two concrete monsters: Friends Arena (home stadium of AIK) on one side and the Museum of America (the mall) on the other. Constructed as part of the same development in 2015, this massive infrastructure seems to be entwined by more than just bricks and mortar. Today, big business, football and consumer culture go hand in hand; it's a reminder that football has changed and its constitutional allegiances have shifted. I remember a meeting with Hans, (AIK's former chairman during the clubs most successful period):

> Hans: The real change for football, of course is the Bosman case (free out of contract transfers), probably that is the time when it really changed, from being a bit more of a traditional sport to being a commercial business and when it's a commercial business it's like everything else you have to get the money, get the money, get the investors. (Field note: formal conversation. December 4th, 2017).

Simon Critchley, Professor of Philosophy at The European Graduate School and avid football fan, puts it like this:

> I'm playfully serious here... Football gives you a paradigm for the kind of dynamic configurations of space and time which I think are constitutive of wider macro social processes and what football makes particularly evident to me is this fundamental dialectic that it has at its core: The form of football is association, which I extend a little bit to say its socialism and collaboration, and the material substrate of football is money and not just any money the dirtiest most horrible money that you can imagine. From the most questionable sources (Field note: Online lecture 12th September)

Footballs dirty money is evident throughout the game (for many, FIFA is now synonymous with corruption) but I wonder if this insidious influence was tempered by the actions of Swedish Sport and football fans? Hans explains:

> Hans: Between '97 and '99 a big analysis was made at the Swedish sport organisation (RF)… we thought that it would've been better to set the rules that you allow 100% for the market. But that was not the case… so the rule in Sweden became that the traditional club must have the majority, the voting majority.
>
> James: What did you see as the advantages of 100% market?
>
> Hans: The possibility to raise more money from the market… At that time, I was quite optimistic… it won't take so many years until we can change this… and it's still the same, 17/18 years after. And today there is no debate.

James: Is that because it's working?

Hans: No I wouldn't say that, it's a mess (smiles), no not really but of course this is the key issue for the strong supporter groups in the big clubs; AIK, Djugården, Hammerby, Goteberg, Malmo for the big supporter groups and the hard core fans this is the big issue and until today they have won this fight. There is not so much strong management and strong forces in the club to change.

James: Why do you think they (the fans) want to keep it the same?

Hans: Many reasons, to keep the power and to show who has the power.

James: What do they see as the risk if they were to go 100%?

Hans: Private owners or big investors would come in and have the majority… and they (the fans) always talk about the fact that they (the owners) will take decisions that will not be popular, raising ticket prices, they talk about the situation in England with the Premier League clubs they have changed the group of spectators from the real supporters to the 'Thai people'" (Field note: formal conversation. December 4th, 2017).

Economic exclusion at football grounds has been evolving as far back as the year 2000 when Roy Keane lamented the 'prawn sandwich brigade' at Manchester United in England:

> Away from home (Old Trafford) our fans are fantastic; I'd call them the hardcore fans. But at home they have a few drinks and probably the prawn sandwiches, and they don't realise what's going on out on the pitch
>
> (Field note: BBC website Angry Keane slates Man Utd fans. 9th November 2000)

The term 'prawn sandwich brigade' became synonymous with people attending football for the luxury boxes and corporate hospitality rather than, as Keane puts it, knowing what's going on out on the pitch.

3.2 'Balls' over talent

Jorge Valdano describes a similar situation in Argentina whereby the game itself has become secondary to other events:

> At the same time a passion for football was overcome by a passion for a team, as if a society that has become ever more individualistic needed something to reconnect it with tribal feeling. Turning clubs into mini nations constructs an identity, a community that must be defended as a matter of life and death. In the stands violence took over; on the pitch, we said

goodbye to the olés and welcomed in a world where huevos – balls – are more important than talent.”

(Document analysis: Guardian article: What’s wrong with Argentina? We now value ‘balls’ more than talent, 25th June 2018.)

The emphasis on ‘balls over talent’ is evident at the top end of Sweden’s largest youth club; Brommapojkarna (Bromma boys or BP) according to a national newspaper:

It was Aftonbladet who revealed, on Friday afternoon that Broma boys' coach Luis Pimenta controls the team through threats, harassment and pure bullying. Several players anonymously described a leadership based on a macho culture that violates everything Brommapojkarna stands for, as one of the country's largest youth clubs.

After the big loss against Häcken away of 0-6, Pimenta has forced the players to stand up for the others and talk about situations in the match when they played like a "pussy" and when playing with "balls". Throughout the season, Pimenta has also had a hierarchy list on the wall where he ranks the various players.

(Document analysis: Dagens Nyheter article: Efter anklagelserna: BP:s tränare tvingas ta time out, september 2018. Translated from Swedish)

3.3 Disturbances and disorder

While Old Trafford’s atmosphere was in decline throughout the early 2000’s, the atmosphere at Råsunda (AIK’s previous stadium and spiritual home) was intensifying along with the reputation of certain factions of AIK fans:

Hans: We had a lot of disturbances during matches and disorder and this was highlighted in the media… big stories in the media… problems with supporters… were big stories… in Sweden, but today it has changed, today it seems to be more accepted that this is not only a football problem, or a club, or the event itself, it is more a mirror of the situation in the community.

At that time it was more that this is your problem, tell us how you are going to deal with it… but it’s tougher today, there was not such a big influence in the club, that was a change sometime around 2004-2006… the hardcore fans … errr came to play much more of an active role within the club.

(Field note: formal conversation. December 4th, 2017).

Pitch side disturbances are not resigned to the history books, with fighting evident at a derby game against Djugården in the 2017 season. Within the club frustrations grow and as these incidents continue and, alongside these frustrations' doubts grow around the collective intention to change. Leaving the Djugården derby after witnessing the fighting a colleague (at AIK) commented:

> Mark my words! There will be nothing about this on the (club) website come Monday... I guarantee it; they are too weak to say anything. (Field note, informal conversation, 27th August 2017)

For some the frustration is more personal than others. Some staff at AIK are harassed and threatened by hardcore fans leading to ongoing investigations involving the police.

> I'm tired of being chased by people who go after my family… I can live with them doing that if the club stands behind me, but they don't.
>
> I'm not even sure that the firm want us to quit working (the way we do) they just don't want it to be me because I'm doing quite well and then… I'm an enemy for them.
>
> I mean Sofia… she got a knife in the door… the environment in AIK is terrible, like this is an interesting club to work in but you don't want to be here too long, it eats you up. (Field note, informal conversation, 7th December 2017)

I recall a conversation about the ongoing harassment of AIK staff and in particular the mindset that fosters pitch side disturbances and off field persecution:

> Jasper: People seem to think 'Monday to Friday I'm an accountant, or I work with children but on Saturday and Sunday I just spread hate… I mean the people I've met who engage in those types of activities, I mean they're normal people.
>
> Erik: That's the fucked-up thing.
>
> Jasper: That's how it is, Monday to Friday they are just like you and me and they seem to share the same values and then Saturday, Sunday or Friday after 6 they just forget…
>
> Erik: I actually want to sit down with them and ask why?
>
> Jasper: Well I mean that kind of behaviour is quite normalised in the culture that I grew up in, what a lot of my older friends would call "Swedish" culture. It's the same rhetoric used to defend Swedes going abroad and getting stupid drunk and vomiting all over someone's porch, just because its Cyprus its fine; and it's the same 'well you've got to relieve yourself, I'm on holiday, or its the weekend, I can behave however I want…' No you can't it's the same basic

kind of morals and ethics that can be applied on a Saturday as on a Thursday... and the people you meet every day deserve the same amount of respect and the same social rules and laws apply but they just don't see that… they can relate to laws or rules and expectations regarding their behaviour in the context of their work place, like I'm at school now or whatever but as long as they are not (in that context) they can't really accept any kind of set of values…

(Field note: informal conversation 15th September 2017)

3.4 Social responsibilities?

A tension seems to centre on AIK's social responsibilities. I remember a conversation with Todd, a founding member of one of AIKs hardcore fan groups, The Black Army.

> AIK shouldn't be… the bad conscience of society, you know. Oh, the integration doesn't work; ok we'll take care of that…
>
> We are a football club first and foremost. Let's create good football players… and let's make society create good people. I know they go hand in hand, AIK is part of society, absolutely but if you just want to categorise…
>
> (Field note: formal conversation, 23rd November 2017)

This tension bleeds over into seemingly unrelated areas; I remember Dave (a fellow academy coach) showing me the photos of our academy coaches' Futsal team (on Instagram); we each had a mug shot in AIK kit… but one photo was missing? Ben's photo had been 'taken down' because he was wearing the rainbow coloured Captain's armband (see Figure 25). I asked why? And was simply told the 'firm wouldn't like that'. (Field note: Informal conversation, 16th November 2017). I wonder if "all the (football) clubs in the Swedish first division will wear them this season" (Field note: informal conversation, 25th of March 2017) if our social futsal team can't?

One member of staff uses an interesting analogy to explain the spreading influence of some of the fans:

> Erik: This is actually how I see it… if this is AIK (he draws a picture of a stick man) then the firm and some bad people are like a tumour (draws a dark area on the arm) and by working with values, our job is to stop the reproduction of cells. Quite simply they are cancer and we need to stop the cells reproducing… but like a disease they are deciding what the person can do… (lowers voice too much and its inaudible)… they are really like a disease because we can't do anything because they are making the rules.
>
> James: The first thing is knowing the disease is there and its moments like these that you really know it's there and to what extent it's there.

Erik: Yeah like you say, knowing you have the disease and accepting that you have the disease… I think that's the step not everyone has taken… because before they can do the surgery, we need to be on the same page… or we'll die, if we don't do anything about the disease.

(Field note: informal conversation 15th September 2017)

Tensions in ideology and identity may be best surmised by the man who knows them best: Edvin Andersson has been an AIK member for a long time and has worked as the club's youth sporting director.

Edvin: Sponsors, other clubs, general opinion from AIK supporters and other people all over Sweden; the picture is of a dark threatening club, connected with violence … I want AIK to be light and welcoming and an including club for everyone to be in and that has not been the case for many years now, since they (the hardcore fans) got too much influence… (Field note: formal conversation, March 14th, 2018).

The depth of this influence became apparent as our conversation continued:

Edvin: They (the hardcore fans) wanted to have influence into how AIK make decisions about who is to be the coach, who will be the sporting director. Actually, I think they were aiming at, and they also got power of the club, in all perspectives…

The nominating committee (for the board) had to listen; they have had to have special meetings with certain fan groups… (Field note: formal conversation, March 14th, 2018).

I recall more of my meeting with hardcore fan Todd and his take on the fans' role:

Todd: We should be members of the club because if you're not "what the fuck are you doing there" which also means that you have a responsibility for the club to make sure that it's developing in the ways that a football club, a sports club in this case should.

And so that's why there's a lot of influence in what's going on, what's happening and why it is happening… because we've seen a lot of "players" coming in and I say "players", as not just physical people – on the pitch players, but "players" around the club coming in and either wanting to take the limelight or make a personal gain… by reaping from what other people have sown and not giving anything back to the club. However, I have no problem with people coming in and being successful, even for your own personal gain brilliant. If you become the next super-duper mega professor in football, excellent, brilliant then I know AIK has been

given the benefit of that... during the journey. Basically, it's like a salesman. The best salesman is the one that works on commission because if the salesman becomes a millionaire, brilliant because that means the company they are working for are mega-millionaires.

James: Is that in regard to (physical football) players as well?

Todd: Actually not so much anymore because you don't go to a Swedish side for the money, you don't, because, I mean you don't have to go to any extreme, you go to Norway or you go to Denmark if you want the big money. But we see that some players have come to AIK and they have been given the education and the lift in AIK and then you leave as a Bosman (free transfer). Whereas others (professional players) have said I'm signing a contract for another 2 years but you're selling me the day after and at least the club will get some money for it.

(Field note: formal conversation, 23rd November 2017)

The influence of non-playing 'players' reminds me of the clubs use of SMART goals; essentially cones with flashing lights they became a running joke among some coaches.

James: Do you think there is a bit of a problem, with SMART goals, with people in the office thinking they are good… like a knowledge gap?

Carlos: Yea that's exactly what it is.

Pontus: They see a product that looks good… they see… a complicated system trying to control complex systems.

(Field note: Informal conversation, 29th August 2017)

I wonder how SMART goals got introduced and became accepted within the club? I wonder if they represent someone using AIK for personal gain?

3.5 Everyone's Club

As I continue to walk away from the mall, I start to see AIK's iconic club crest appearing in the shop windows, billboards and on flashing screens. AIK stands for 'Allmänna Idrottsklubben', which roughly translates to 'The Public Athletics Club' or more broadly 'Everyone's Club'. The name suggests an inherent emphasis on inclusion, or as Todd described it, non-exclusion in the stadium and on the terraces:

Todd: I think the North bank or the terraces on the whole is one of the most non-excluding places that you could ever go, as we usually say "when you come here you have two things to worry about, black and yellow". I don't care what your political beliefs are; I don't care about your religion, where you come from, who you go to bed with etc etc. I don't give a 'beep' just

support the team and let's have a drink together because we won or we lost, we might not be best friends, but we respect each other, where else in society would you really get that…

(Field note: formal conversation, 23rd November 2017)

However, at a recent Allsvenskan (Swedish top division) game against Göteborg, a fan wearing a pride (rainbow) T-shirt (in the north stand) was told he'd 'better zip-up his hoodie' because people around him didn't like it. The incident was recounted and discussed on social media and I remember talking to Jasper about it at the office. Jasper works closely with the sporting director of AIK's youth set up and has been involved as a player, a coach and grew up in a family of AIK supporters.

Jasper: I think AIK has had a lot more political tension over the last 30 years… (compared with other Stockholm clubs) one of the reasons for that is that it's (the club and its fans) so much more diverse, so it has a lot more extreme parts. I mean there are extreme parts in the other clubs too but not as many and maybe not in both directions... I actually think it's harder… to be a Nazi and to be an openly Nazi guy standing with your Nazi mates if you're supporting Hammerby and I think it's harder to be an openly left wing extremist and support Djurgården but in AIK its evenly matched, and in a philosophical sense its good because AIK is for everyone, but in practice it's super hard because the common denominator becomes harder and harder to find, especially for them because they don't want to see that they have anything in common with the next guy…

(Field note: informal conversation 15th September 2017)

Over time, fans have re-prioritised their loyalties, this is captured by the order in which they prioritise a) the immediate supporter group to which they belong b) the terrace on which they stand (e.g. the north stand) and c) the club itself (AIK).

Jasper: I've heard different supporters say you have to have your priorities straight and there are three things that you need to prioritise and what values you have as a person determines in what order you put them, and it's; 1. Cliqen, 2. Klacken, 3. Klubben.

James: Some people think club is first… ?

Jasper: Exactly, that's how it used to be, but I hear this from a lot of older AIK supporters… the general feeling is that the order has changed from Klubben first (AIK first) to Cliquen, Klacken, Klubben, which for them, is super weird and wrong…

Historically the biggest supporter group is The Black Army; the group Todd co-founded.

Jasper: If you see old images of black army away games in the '80s and '90s you see a lot of

graffiti and a lot of anarchist 'A' symbols and wrecked cars and people throwing up, you know mayhem…but you also see black people, white people, children, pregnant women… a huge mix which was even more diverse than it is today…

The polarisation has come (today) and the female supporters have the female supporters' club… and in Sol Invictus you have more immigrant supporters and Ultras Norde is younger and whiter… its interesting because its more about clubs (supporter groups), cliques and super specific divisions now… compared to back in those days ('80s – '90s)…

I'm left wondering if fan groups atomising into smaller and smaller constellations (or perhaps polarizing into smaller and smaller groups) mirrors the world's movement towards reductionism, individualism, extreme competition, self-interest and political polarisation? Is it the continued evolution of the pattern identified in Argentinian football by Jorge Valdano:

A society that has become ever more individualistic needed something to reconnect it with tribal feeling. Turning clubs into mininations constructs an identity, a community that must be defended as a matter of life and death. In the stands violence took over; on the pitch, we said goodbye to the olés and welcomed in a world where huevos – balls – are more important than talent.

(Document analysis: Guardian article: What's wrong with Argentina? We now value 'balls' more than talent, 25th June 2018.)

However, Jasper continued, explaining a counter movement:

Jasper: Have you heard of ASK (new fan club/group), they were founded 3/4 years ago, when we moved to this arena and their point was to gather all the supporters, so you can be a member of all the other groups and belong to this one too... just like AIK, we are for everyone… and they spend a lot of time, what you might call lobbying, effecting politicians around the pyrotechnics bans and writing articles… their former chairman has a doctorate from KI he's a microbiologist and he's been involved a lot in the national debate about Bengal flairs… 10 years ago that organisation could never have happened because it goes against the core belief system of the normal AIK supporter… like to organise is not really in our blood, we like to have 'black army' (historically the biggest supporter group) or some club where we can say who are we, 'well we are black army' but what is the black army? You don't know (says smirking) because we don't know, and that's the point there is no definition of what the black army are…

In recent years certain fan groups (mostly those wanting to emphasis the value in 'balls') have been louder than others, however this year a greater proportion of 'everyone' (AIK members) raised his or her voice, as dark clouds circling the club failed to clear. Prior to the 2018 AGM, a club member proposed an alternative board and chairman and over seven hundred members signed a petition supporting this alternative. And at a meeting that regularly seats 30 people, 924 members turned up to vote. Edvin explains it like this:

> We have approximately 17,000-18,000 members and 924 attendees at the AGM was an all-time high, so if you ask: Is the member democracy working well? Yes, it is working well because the alternative (board and chairman) was voted in… I think two-thirds voted for the new chairman.
>
> I have a vaguely positive feeling, I'm not sure… there is a polarisation between the different groups of supporters and the hardcore supporters are not satisfied (happy) with the outcome of the meeting so I think they will… act. So, we will see… we'll see, things happen in AIK and I think chaos is the normal situation, in some way.

3.6 Smokinglir

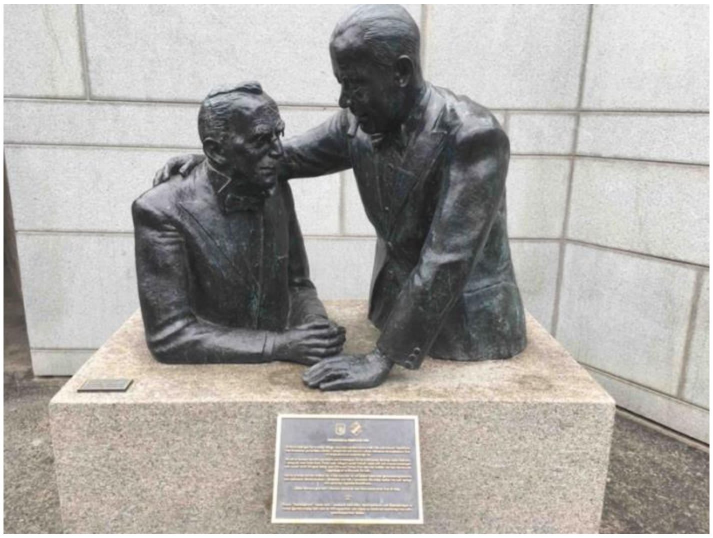

Figure 25. Smokinglir statue outside friend's arena and the mall of Scandinavia

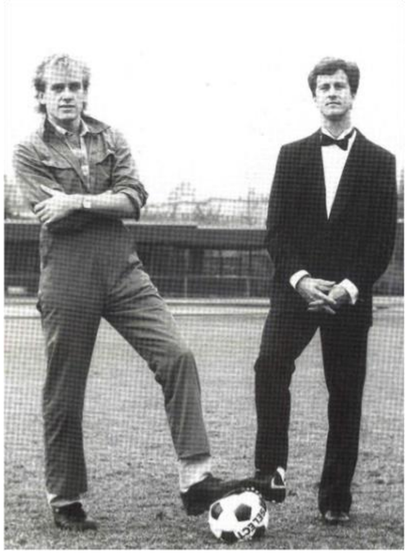

Figure 24. Smokinglir med skruvdobb

As I continue to walk in the shadow of the mall, I see the AIK Smokinglir statue (Figure 36); it shimmers as a rare beam of Nordic sunlight cuts between the overbearing buildings. Dwarfed by the mall behind it I'm struck by one great institution sitting in the shadow of another. Funded by the fans who purchased limited addition match shirts, the statue is called 'Smokinglirare' and the plaque reads:

> "The public sports club is celebrating 60 years and at one of the tables we can see the club's

founder and first chairman, 83 year old Isidor Behrens in conversation with Peter Kock who had played football for AIK and the Swedish National team and won the national championship in 1923 as well as the Olympic bronze in 1924. But this night he was present as the chairman of AIK. What they spoke about we don't know but it was a meeting that transcends generational boarders and defines sport in general and specifically AIK. A meeting between two ordinary, yet legendary AIK people"

I remember the first time 'Smokinglirare' was explained to me, it was the first time I went to Karlberg (AIK's first team training complex), the first time I attended a Forum Karlberg (the monthly educational evening seminar) and the first time I met Sven.

> When I arrived, Sven was sitting with Stephen and gave me a warm welcome. They were looking through a presentation about the club, its history and identity, we spent some time talking about a specific picture of 'the smoking player with studs' (see Figure 36)... Sven took me through the dressing room and talked me through each and every player, giving me the year of their birth and a little on where they had come from. When he got to Stefan Ishisaki he said excitedly 'Ishisaki is an AIK player, a pocket player'.
>
> (Field note: headnote 22nd March 2017).

Smokinglir (smoking play) is re-emerging as AIK's football ideology; smoking players play football as if they are wearing their 'smoking suit', 'dinner jacket' or 'tuxedo'. They are noble but humble; they play with elegance and trickery. Originating in the early 1900's the idea evolved during the 1960s and 1970s to incorporate 'smokinglirare med skrivdobb': The smoking player with studs. Representing the working-class background of many AIK's fans. Sven explained that the stadium is at its loudest when the players are 'flying into tackles' or 'tricking (deceiving) the opposition' – the smoking player is a humble hard-working maverick. In this matter the fans seem to agree:

> Todd: Another great example, but here you have an elegant nature to it is Ivan Obolo… every time the defender of the other team played the ball back to the goalkeeper he was there chasing it down, because you never know and sure enough the goalkeeper gets stressed. Especially here… if you're in front of the north bank and he's chasing you down, of course you'd be nervous…
>
> So, he was an absolutely elegant player… and the same heart and passion as Kenny Pavey and you love them both equally as much but for very difference reasons. It says a lot when Hammerby and animal farm (Djugården) supporters go 'Jesus we'd love to have him in our team, we hate them, but we'd love to have them'. So that's basically what spectators are looking for.

(Field note: formal conversation, 23rd November 2017)

Recently, Sven uncovered the nuance between smoking 'player' and smoking 'play'. Talking to one of the club's oldest members Sven was told that the original ideal was not so much a celebration of an individual's trickery, but the team's collective creativity. However, as time has passed, the idea has drifted towards an emphasis on the individual, perhaps another example of a neoliberal drift.

3.7 Tournaments

The statue disappears out of sight and I arrive at the office for a meeting about the Lennart Johansson Academy Trophy (LJAT); a tournament hosted annually by AIK with clubs attending from around the world (FC Barcelona, Athletic Madrid, Anderlecht, West Ham, Arsenal, Inter Milan, Vancouver White Caps, Besiktas and Everton to name a few). Andy is the main man in charge, and we talk about the unbelievable behaviour he witnessed at an Under 10 grassroots' tournament over the weekend.

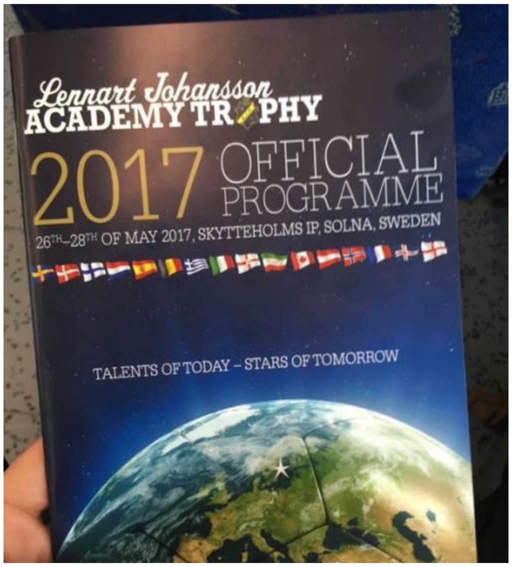

Figure 26. Lennart Johansson Academy Trophy (LJAT) program

Andy: The younger teams still have many parents who think their son should be the next Zlatan… when we have incidents with parents, it's at the younger ages.

For example, at the tournament this weekend… On Sunday there was a team and they played a game (boys from2007, ten years old), they lined up to start the game, and suddenly a father runs onto the pitch, takes his son from the bench and puts him in the line-up (out on the field)… Takes the coach's son and puts him on the bench, so the coach and the parent start arguing and almost fighting…we don't have any gold medals or results even and still this happens.

James: No league tables, no finals?

Andy: No they are too young… and I think this wouldn't happen if the boys were 15… but they are so young and the parents are like… they don't have time to wait anymore, he needs to be the best now and he's only 9 or 10! It was horrible. I was so ashamed... (Field note: formal conversation, December 7th, 2017)

Andy transitions into a story from the LJAT three years ago:

Andy: "There was this coach from AIK and he got upset because he thinks the referee missed

a penalty and he starts yelling at the referee for the last 20 minutes of the game. AIK lost the game and once the game was finished … this AIK coach, chased him (the referee) into the locker room, the referee had to lock the room and the AIK coach was banging and smashing the door and he almost crushed the door. He was banging it and yelling 'come out you coward'… it was horrible… I felt ashamed because AIK didn't do anything about it … that coach is today in the first team.

(Field note: formal conversation, December 7th, 2017)

After this incident Andy arranged for UEFA qualified referees to oversee the tournament and today UEFA and FIFA use the tournament as an educational event.

Andy: So that (the incident with the AIK coach) was actually something really, really bad but in the end, it worked out really well for us…

Why don't we have incidents anymore? … The main thing I think is that AIK communicates much more about fair play and equal values and good soft values, rather than just (being) focused on hey we need to pass the ball better, that's actually the second most important thing; the most important thing is to develop people… and when AIK coaches see okay, clubs like Barcelona, Arsenal they don't fight with the referees… and this is what LJAT is good for … because AIK coaches can see how it is in Solna with the professional coaches (Field note: formal conversation, December 7th 2017)

As Andy talked about more incidents he asked:

Have you heard the story of how AIK Stilen came up?

In the team (born) 1990 … there had been a really big incident with boys (born) '88… the older boys were harassing them sexually and the scandal…was big news in every magazine in Sweden… So AIK Stilen started and… the pilot was my team (born) 1990…

Instead of some training… we actually had education with Jussi (on teamwork and social cohesion)… so he worked twice as much with my team (born 1990) as with the other teams and still I think now 15 years later… that team has developed more players than any other team today in AIK; to the national team; to the higher league, from those 20 players when they were 14, I think like 7 players were in the U19 national team.

Like Daniel Sundgren … Magnus Eriksson, Viktor Lundberg etc and a couple more, so how could we develop those high-level players when we didn't have an academy.

(Field note: formal conversation, December 7th, 2017)

Andy describes how instructions from the club and parental support helped shape the environment:

> The instructions from AIK was like okay, you have 30 players and you have to give every player a chance in the number one team… and we didn't hear anything from the parents… the parents they were like really, really good and I think it was because some of the parents were former players … and the other parents were like okay, if that guy doesn't complain why should I complain…
>
> Everyone had equal playing time all the time and I started with different line ups every game, how could they manage to actually reach quite a good level, and I'm not saying it's because of me, no, no it was because of the parents, it's the environment…
>
> I actually see the other guys playing in that team (born 1990) and they have actually been quite successful in business and in work you know, so we did something well already back in that time…
>
> I think…what we have forgotten, is to work with the good values, like we did five or six years ago…
>
> The incident we discussed at the start of the meeting at the LJAT when the coach ran (after the referee) – no one in AIK actually cared… I mean what! People said, 'the coach was bad, so what' I mean that attitude has to be changed and it has lately, but we still need to change more…
>
> (Field note: formal conversation, December 7th, 2017)

Even in the short time I've been at AIK, it's noticeable that things are changing but it's also evident that change is needed, both on and off the football pitch… I remember watching AIK's youth against FC Barcelona at the 2017 LJAT and hearing the tongue in cheek comments of spectators; "It's like two teams playing a different sport on the same pitch" (Field note: informal conversation, 26th May 2017). While it may seem unfair to compare AIK and FC Barcelona, our Danish counterparts from FC Copenhagen gave Barcelona a great game in the same tournament (in 2017). Perhaps there's a significant gap between Swedish and Danish football? I'm left wondering why?

3.8 Part 3 Summary

In the first section of part 3, I introduced the theme Bosman and Business in conversation with Hans (AIK's former chairman) and highlighted the global recognition of football's 'dirty money'. Hans describes how Swedish fans resisted private ownership of clubs and stalled the economic exclusion increasingly evident in the UK. Documentation is then used to introduce the interpretation of a

worldwide trend, whereby the game has become secondary to corporate hospitality and ‘defending the club’s name’ with violence in the stands.

Examples of threats are introduced through informal conversations before Jasper gives insight into an attitude of escapism – a desire to escape social responsibilities, rules and expectations – that has become so pervasive it becomes “quite normalised in the (Swedish) culture”.

The next section continues to highlight a tension around AIK’s “Social responsibilities?” Tensions are surmised by the youth club’s sporting director and are evident in Todd’s use of a salesman analogy too, which implicitly reinforces the tenets of neoliberal capitalism; an economic system predicated on personal gain, with little emphasis on ‘giving back’, the very thing the fans are rallying against.

“Everyones’ club” continues the discussion on AIK’s social tensions but introduces political dynamics too; the section highlights co-adaptation of social groups in response to one another. Finally, the club’s AGM and changing board are discussed in conversation with Edvin, who highlights a polarisation between the supporter groups and recognises that “things happen in AIK and I think chaos is the normal situation, in some way”.

The section “Smoking play” moves the narrative closer to the football pitch and highlights the clubs re-emerging football ideology. The section titled “Tournaments” begins by describing extreme behaviours and expectations of parents and coaches. Andy also describes the incident that led to the formation of AIK stilen, the clubs code of conduct. Throughout Andy’s discussions there are examples of (co-adaptation) extreme events promoting extreme reactions and therefore there is always a dynamic, always change, another theme. Andy also suggests “I think…what we have forgotten, is to work with the good values, like we did five or six years ago…” This section concludes by highlighting a need for change both on and off the pitch.

4 Part 4

English & Italian influences

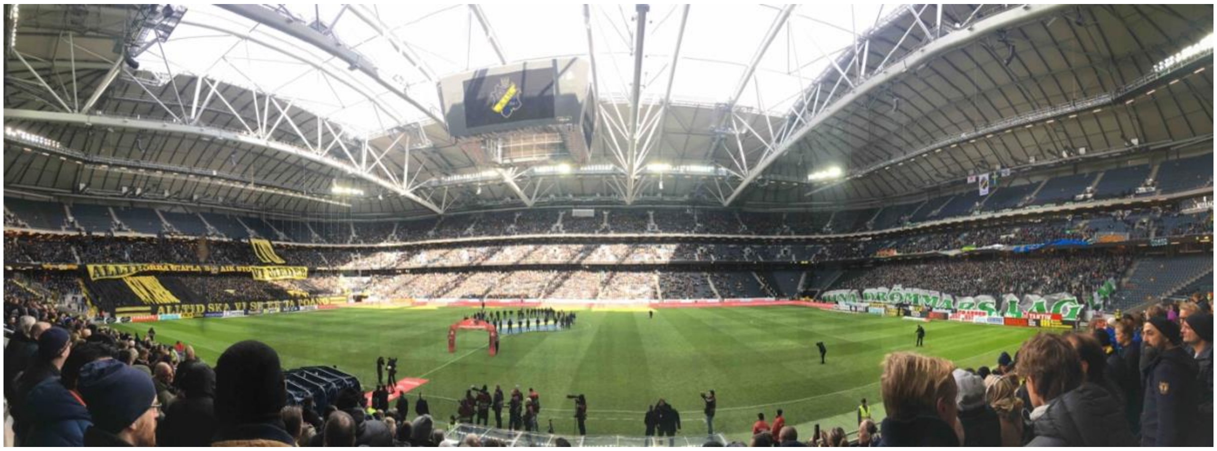

Figure 27. Inside Friends arena in the build-up to the derby against Hammerby

I leave the office and walk to Skytteholms IP (sports field), known as SKIP; SKIP and RIP (Råsunda IP) are the sports fields where smoking players are taught (or learn) their trade. I arrive early for a meeting and a discussion on player development between the club's academy coaches and development coaches. After some initial housekeeping, the talk turns to interpretations of football:

> Sam: The problem is the old type of game that Sweden played when we were younger, I was always taught to play a long ball and chase after it and back again and counter-attack. All the time and when you are getting older you take the old style with you and coach the kids, so you just pass it on instead of learning the new stuff… instead of having new knowledge and passing it through to the kids… I think most of them, not all of them, don't have a clue about soccer"
>
> (Field note: informal conversation, 23rd August 2017)

I cringe at the use of ‘soccer’ and start considering the scary similarities between this description of football and my childhood experiences (in the late 1990s) in England. I recall Pontus providing a possible rationale:

> Pontus: You had Roy Hodgson and Bob Houghton come here with Halmstad and Malmo and they were playing 4-4-2: a very strict style of English football… Malmo got to the European Cup final (now the Champions League) against Nottingham Forest in 1979 so… people wanted to go the English way. (Field note: Informal conversation April 28th, 2017).

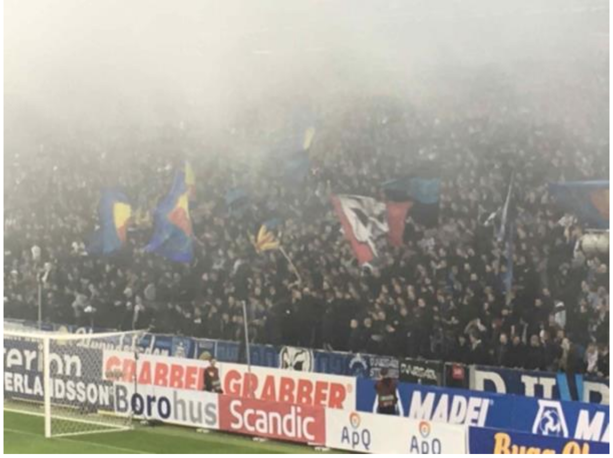

Figure 28. Union Jack flown amongst the Djugården fans

Bob Houghton arrived in Sweden in 1973, the same year that Johan Cruyff moved to FC Barcelona to play under Dutch coach and ‘total football’ pioneer Rinus Michels. I’m struck by the historical significance and lasting influence of these moments in time (chronosystem). F.C. Barcelona (and eventually Spain) evolved Cruyff’s vision: often referred to as the positional game. While Sweden remained fixated with English approaches on and of the pitch. As Todd explained to me:

> Todd: Swedish Television televised one English game a week on Saturdays… it was like going to church basically; it’s Saturday afternoon football and we are watching it… and that meant a lot of people flew over to the UK mostly to follow their particular (English) team and we had a lot of British influence… A lot of British influence on the terraces.
>
> (Field note: Formal conversation November 23rd, 2017).

Englishman Stuart Baxter is revered as one of AIK’s most influential coaches after winning the championship in 1998; in a season that started with six 1-1 draws. Edvin explains Baxter’s influence like this:

> Edvin: Lots of his ideas about football and about how football would be played went through to Mike, to David Eriksen and to Barry (current first team coach and sporting director at AIK) so they are building in the same direction (Field note: Formal conversation, 14th March 2018).

An innovator at the time it has been suggested that even Baxter felt constrained by the strict style of English 4-4-2 gripping Sweden and AIK. A long-time coach in AIK gave this example:

> He (Baxter) played with 3 central midfielders and one high winger with the strikers starting on the other side. Like a 4-3-3 but no one could say that because in AIK we play 4:4:2 (laughing)…
>
> (Field note: Informal conversation, 18th March 2018)

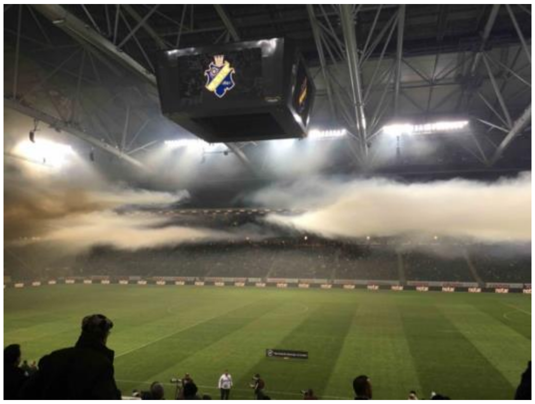

Figure 29. Smoke from flares delays the start to the derby

However, Sweden's football and fan culture are not limited to English influence.

> Stephen: Italian football was broadcast on Sundays. So, the English Premier League was on Saturday at four and then you had Serie A at three or later in the evening on Sunday.
>
> James: What's the Forza thing?
>
> Stephen: It's the flares and the smoke and the huge flags.
>
> James: So that's where the fans got the (the Italian influenced) flares and the flags?
>
> Stephen: Yea… I think the Swedish fans have taken it really, really far, probably beyond the Italian fans, at least today. At least the bigger clubs…
>
> (Field note: Informal conversation April 28th, 2017).

To the frustration of many fans and club officials, the start of AIK games are often delayed as flares create clouds of smoke in the stadium (see Figure 40). As well as influencing the questionable behaviour of the fans off the pitch, the Anglo-Italian emphasis is also evident on the pitch.

4.1 Playing style

Many A-team related Forum Karlbergs (coach education evenings) have been spent discussing the defensive concept of 'shape'; essentially, a passive defensive tactic (a block) to prevent your opponent from advancing; something Italian teams are historically renowned for. I remember one of my first experiences at Forum Karlberg:

> "The forum started with Sven talking the coaches through the 3:5:2 formation and then asking each coach for a brief report on what they are working on… Although I missed a lot of the detail… I remember thinking that the academy teams where imitating the first team. A table

was drawn up on the white board, a row for each academy team and one word was repeated at almost every row: shape" (Headnote: 22nd March 2017)

The memory fades and I'm drawn back to the conversation at hand, a conversation led by Dan, one the club's development coaches; Dan is quintessentially AIK. In his family, you're born into AIK, he once told me that the first thing his Dad did when he found out he had a grandchild was sign them up as an AIK member. Dan also ensured that I was aware of the correct Swedish pronunciation of AIK (ahhh-eeee-kor) while we were away at a tournament together. In the meeting Dan is saying:

> Dan: Swedish people are not so open… I was just about to say I think James also taps into something that is really very Swedish you know, the fact that we as a people, we don't stand out, that is why Zlatan has been so controversial! Because he stands out… (Field note: Informal conversation 25th April 2017).

I remember reading Zlatan Ibrahimovic's book. As a young 13-year-old he had started out at Malmo FF, the club that had epitomized strict 4-4-2 football (under Bob Houghton) and reached the pinnacle of European football in 1979. I remember Zlatan's "take" on coaches and tactics:

> Your supposed to respect the trainers… Or, more accurately your supposed to listen to them and learn their stuff, zone game, tactics, all that stuff. Yet, at the same time don't listen. Just carry on with dribbling the ball and the tricks. Listen; don't listen. That was my attitude.
>
> (Document analysis: I Am Zlatan Ibrahimović)

Dan continued to describe the typical Swedish mentality:

> We work as a collective we work as a team, we are no better than the next guy, we work hard and I mean that's something that we get with the Mother's milk... So that is something that defines us as a people… Of course, that taps into everything we do in school and in football, work hard in everything, so … yea the Swedish mentality (laughs) it has an underlying structure for the whole thing absolutely… (Field note: Informal conversation 25th April 2017).

The coaches start to talk about how this 'Swedish mentality' or 'structured approach' might translate onto the pitch:

> Dan: I've been involved for like five years now, the information we got for the youngest players when we start, the training companion (information-training book) we got from the club… everything is forward; throwing, moving forward, don't train passing just train to take the ball and move forward with speed… but this is also what we have been giving new parents and coaches and it's been really very much throw forward, go forward take the ball with you forward, move the ball forward.

(Field note: informal conversation 25th April 2017).

I'm struck by the similarities between this emphasis on always moving forward and Stephens's explanation of wider socio-cultural-historic influences:

"We come from a Newtonian paradigm which in itself is very linear, then we have built all our welfare through our industry… I think we relate success to planning and linear models in general…

However, as Stephen points out, this might not be ideal for football;

… but maybe since football is dynamic and unpredictable maybe we need to question that, because of the nature of the game, not because we want to be someone else but because of the nature of what football is…."

(Field note: informal conversation, 5th May 2017)

The memories of my first AIK holiday program flood my mind; I was coaching with Jake and we were shocked by the linear pattern of play during the matches. Eleven and twelve-year-old players clustered around the ball like bees around honey seemingly obsessed with only moving forward. If a player happened upon the ball, they ploughed forward making a beeline for goal with little appreciation of spaces or gaps. Depending upon the manner in which the ball arrived, the forward motion manifested as a 'big kick' or often a bumbling, stumbling dribble. Like an endangered species, passing was scarce. The game took on a formulaic tendency; a linear pattern of dribble forward…get tackled, fight for the ball, lose the ball and then the opposition repeats the method; dribble forward, get tackled, lose the ball and repeat and repeat and repeat. It was almost as if the players had blinkers on; constrained or perhaps controlled to only see forwards: Attention attracted by ball and goal, nothing else.

This one-directional focus also became increasingly obvious at a recent academy Under 10 tournament in Berlin. As the games progressed and the intent on winning intensified, AIK's young players seemed to suffer from an increasingly severe case of tunnel (reduced) vision. Almost every AIK throw-in was hurled forward into a pack of players thrashing around like piranha. I remember AIK's head of academy turning to me on the sideline and questioning why 'they never throw it back to the goalkeeper?', the player with the most time and space.

Any intention to utilise time and space – the emphasis of many training sessions during my time in the academy with this team – evaporated as the ball was feverishly booted, or thrown, forward to cheers from parents. Our players also started pulling opposition shirts and throwing arms and elbows, some tackles started to resemble rugby more than football. At this point, even if players were brave enough to try and pass or dribble, they often had no-one to pass too as their teammates were hiding, seemingly anxious of making a mistake that may prove decisive.

Limited attention or awareness seems to be evident in the older academy teams (U17-U19) too, with Jake commenting that:

There is no balance; there is no using the space in smart ways.

(Field note: informal conversation, 15th September 2017).

Jake is a UEFA pro-qualified coach who guest lectured on the Spanish pro license teaching tactical periodisation. He comes from Barcelona where he's worked as a full-time head coach in Spain's Segunda B and studied at The Johan Cruyff Institute. Jake arrived in Stockholm in January 2017 and I arrived in March, we'd both moved from Barcelona. Being new to Swedish football culture and AIK we wanted to learn more and having moved from Barcelona we shared a similar view of football (although his understanding of the positional game is far more advanced). On many occasions, we ended up talking about the problem with simplistic and linear interpretations of football and its effect on coaching and players. I remember some frustrations coming out in our conversations:

Jake: It's not understanding the game… I'm not talking about you need to play with wingers or not wingers… but just understanding football is a complex game… just understanding that, you cannot think that this is fucking chess or whatever. We are talking about people and players that are human beings… so why the hell are you trying to chain them and give them just two options in that position or one option in this position.

James: This is exactly the problem with linear thinking… predict and control… the idea that if we are analytical enough, we can control everything…

Jake: To feel that you are in charge.

James: And this goes beyond football because this is the way people live their lives, this is a dominant world view, this is why we have the AIK identity and it's a bullet point list. You can't put coaching into a bullet point list, there are so many moving parts, it's so complex… But it's rare to find people who are…willing to embrace that.

Jake: Serio-lo (Paco) says that only the coaches that are not afraid of losing are the ones that can develop the game (and develop players?)… Because most of the coaches… are in that environment where you just want to keep your job…

(Field note: Informal conversation, 3rd July 2017)

These conversations often took on a feverish quality, like there was too much to say and too little time, too many examples. On the one occasion we found an afternoon to talk in depth and Jake introduced me to Spanish coach Juanma Lillo (via YouTube). Lillo is described as a football philosopher and regarded as a mentor to Pep Guardiola. Lillo's comments capture some tensions between football ideologies. The difference between two football paradigms, the positional game of

Cruyff and the historically strict, direct 4-4-2 that characterized English football, a style of play whereby the coach predicts, controls and commands his players. From Lillo's perspective:

> "You can't predict human behaviour. You can see that if Messi receives the ball in certain spaces, he's dangerous. But that's conditioned by who he receives it from and when, whether his last move came off or not, what his emotional context is, how the opponents react. Positional play, which I work on, allows you to try to provoke certain situations, for sure. But it's more important that you have the intelligence, the culture, to know how to interpret what is happening, to adapt, to understand, and that you are able to seek the solution that gives your team the greatest advantage"
>
> (Document analysis: The Brain in Spain)

Jake left AIK after 6 months of full-time employment as the under 19's coach, and he now coaches a premier division club in Norway. In many ways, those six months could be characterised as a clash of cultures at the top of the academy, people working from different paradigms: Different ideas of a) what football is; and b) how best to develop players and teams. The paradigm of strict 4-4-2 is very different from the paradigm required for 'smoking play'. Predict, control and command does not cultivate creativity, it kills it.

4.2 The illusion of Control

The desire to 'control everything' is not isolated to AIK but it is evident in AIK. Early in the 2018 season, fans took to social media to voice their concerns about the controlling football of AIK's first team (and perhaps a lack of smoking play):

> The problem is rather that Eriksen apparently tries to control the matches, keeping back the players offensive will…

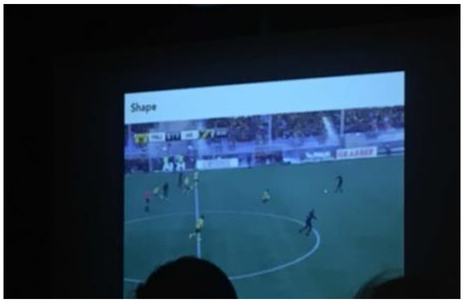

Figure 30. 'Shape' a popular concept

> The primary thing about Eriksen's football is about control. Not to let the game float away. Not to let our players have their own freedoms. Everything should be rigid and structured. As I see it, it's just the diametrical opposite of what AIK needs, to take the next step in its development…
>
> I am quite convinced that this Eriksen passive-controlling football will create significantly greater frustration within the team if it continues.
>
> (Field note: social media post, 21st March 2014)

Unfortunately, a controlling emphasis emerges at junior and youth levels too, after reading about the planned activities and coaching behaviours described in AIK's junior and youth VPL's (yearly plans of activity) Pontus said:

> There is not one VPL in the whole club that I would approve… so much of this stuff is about punishing children.
>
> (Field note: informal conversation, 13th March 2017).

Punishment is perhaps the most recognisable attempt to control people, but I wonder to what extent organizational structures and administrational procedures reinforce or represent the perceived need to control. Recently, AIK's senior team coaching staff presented at Forum Karlberg. The evening was an opportunity for partner clubs and academy coaches to gain insight into what happens in a first team environment. Reflecting on the presentation, a frustrated conversation between coaches went like this:

> Ben: We got a lecture in organisation.
>
> Pontus: Is it a professional organisation or a controlling organisation?
>
> Dave: Controlling.
>
> Pontus: It is the illusion of professionalism through control? That's how I'm experiencing it.
>
> Dave: There is no quality just quantity.
>
> Pontus: Yes, and quantity also gives the illusion of professionalism.
>
> Dave: It's so Swedish to have everything so controlling and to have quantity not quality.
>
> (Field note: informal conversation 22nd March 2018)

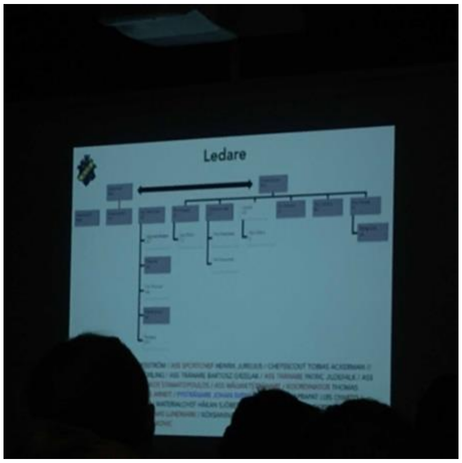

Figure 31. Organisational structure was the focus of a coaching presentation

I recall another post presentation reflection from an ex-AIK first team championship-winning player. Talking about the presentation the following morning he said:

> I feel like it's very individualistic and very controlling and David (Eriksen) is a very controlling person… he always has been... hopefully he knows that and hopefully people are able to tell him. Hey, take a day off...
>
> But you know they get there every day at 7am, every day!
>
> When I see all the staff and all the resources and all the data, I wonder how controlling that

must be, when they say hey, look at this, you can't train today. When we won in 2009 it wasn't like that. People would go out and train and push each other for the good of the team.

(Field note: informal conversation, 29th March 2018)

An obsession with data, order, linear planning, and administration seem to grip many sports organisations, no matter what their stated objective is (for example education). I remember AIK's first research and development meeting this year; we discussed the Swedish Elite Football Federation and how they grade football academies: in particular they give points for development/research projects aimed at improving administration. One group member summed this up:

That is fucking Sweden, points for administration!

(Field note: informal conversation, 21st March 2018)

4.3 In the academy

In AIK's academy, coaches regularly highlight the club's emphasis on administration and organisation:

Johan: When we have coaches' meetings, we speak about organisation not football.

Ben: That is AIK.

Johan: That is Sweden.

(Field note, informal conversation, 26th February 2018)

At least the meeting I'm sitting in now (between the development and academy coaches) is focused on football. However, this does seem to be an exception to the rule; some of the older academy coaches at RIP (academy headquarters) have suggested:

I have never been at a football club where we talk so little about football… At group meetings we talk about bibs, cones, balls, pitches…we talk about everything else… but not football.

(Field note, informal conversation, 12th March 2018)

Jake once described it this way:

Jake: I'm doing all the schedule (working the whole week at RIP) and when it comes to Friday I'm destroyed, even if I'm just spending the whole day at Rausunda doing nothing… but you know, just sitting there and you spend your energy on the wrong things…

(Field note: Informal conversation, 3rd July)

Spending energy on the “the wrong things” seems to refer to lengthy meetings with circular discussions (about administrative procedures and regularly ‘towels’?) that get nowhere near talking about football or coaching. When conversations do turn to football and coaching, they reverberate around “What” to coach, for example what tactics? What session design, what do we need? Balls, bibs, cones! “How” to coach is rarely discussed in academy forums.

This has created some coaches who are truly great administrators but questionable practitioners. Trying to understand the lack of good coaching, one conversation went like this:

> Stephen: They have just given up... It’s because of the coaches before and because of the whole system.
>
> Jake: Is it just that people are… lazy?
>
> Stephen: Not lazy but they are directing their energy to the wrong things…
>
> James: But then it’s also that they are de-motivated… for some of them it will be lazy and some of them it will be de-motivated. It’s hard to tell.
>
> Jake: In other countries, many, many coaches would kill to have that position. I mean full-time coaches!
>
> (Field note: Informal conversation 13th June 2017)

I remember watching the AIK 2003 group preparing for the 2017 LJAT and feeling like I’d stepped back in time. During the main part of the session players waited in lines and passed around cones (without opposition): In no way representative of football, this design is very easy for a coach to command, control, and look competent to those watching.

Talking to a renowned Sports Psychologist (Fredrik) about his time working in professional football clubs in Scandinavia, he rationalised the phenomenon of ongoing poor practice like this:

> Fredrik: The hierarchy in northern European countries is very, very strict so if you pass somebody they are going to kill you… and also like here (Sweden) people know each other and they say; “they have been here for 10 years and we can’t do anything” but he sucks (I say)! He’s such a bad coach, you know he’s destroying your talent, it’s costing millions, of millions, of millions of Krone “ah but you know if I fire him then he has his friend there, and there, and he’s going to be angry”… it’s a mess.
>
> (Field note: Formal conversation 30th August 2017)

The discussion moved from off pitch politics to on pitch practices, that seem to resemble a need to predict and control everything.

> Fredrik: They (club X) have this system (of play) and they start at 10 (years old) following

this game model and it's 'you are #10' (position on the pitch) and you do this and if you do something else then you do wrong, its top down. The learning is so narrowed down, they become an FCX (club) player but they lose all creativity and often the coach is feeding them (controlling) so their self-determination is gone.

Fredrik: And the system is ridged in FCX and its good when we are playing better teams, like Porto last week, when the system is working, we are really good, really hard to penetrate.

(Field note: Formal conversation 30th August 2017)

An example of a player unable to be creative in a rigid system is highlighted:

Fredrik: But now when we put him (a new player) in the pattern he's not the same player, he's actually shit now… but when you give him some free space or he can use his creativity he's actually really good, really, really useful for us...

Fredrik: In the beginning everyone was like "oh fuck he's good" and then yea…"but he can't do this, and you have to do this" and then he starts to think and you see when he gets the ball he's like "no I can't do that, and I have to do that, oh it's too late, what should I do now"… and he's so stressed, he's so stressed being in this pattern and you can really see how he's uncomfortable and then the coaches say…"he's shit…I thought he was good but he can't do anything" and now…we have to buy a new player and this is classic.

(Field note: formal conversation 30th August 2017)

I brood on the impact of game models, systems, and administrative procedures and reporting processes, which may help or hinder. I recall asking some academy coaches about the pressures of their job and where the pressure might come from:

Pressure on Edvin (youth sporting director) puts pressure on the next one, and the next one, and the next one.

(Field note, informal conversation, March 14th, 2018).

Asking if we need a game model (playing system) for 10-year old's I was told:

Yes, when the development boss says you needs to have a game model for 7v7. (Field note, informal conversation, March 14th, 2018).

On top of that some have questioned the lack of relevant communication:

I have ten bosses but no information…

(Field note: Informal conversation, March 11th, 2017)

In the younger academy, pressures seemed to intensify over the winter months and into 2018. Not knowing why, I was left with a lot of questions about our changing practices:

> Why is there more authoritarian coach-centred ‘stop, stand-still’ coaching? Why are players standing in lines? Why is the ball to player ratio so low? Why are sessions becoming more isolated? Why aren’t we emphasizing the values introduced last year?
>
> (Reflexive journal: 18th April 2018)

4.4 On the pitch

After all the development coaches have finished reporting and discussions have died down, the meeting draws to a close and it’s time for the academy coaches to plan the evening’s training session. The complexity of coaching becomes obvious as Ben attempts to plan the evening’s session. The challenge involves combining three age groups (players born ‘06, ‘07 and ‘08) into groups of 4 players so they can play 3v1 in groups relevant to their individual needs. The groups need to be developmentally appropriate and provide some players the challenge of “overload” (playing against older players) and other players the opportunity to “dominate” (playing against younger players)… just as the groups are finalised Ben’s phone “pings”… a text says we have one less player and our planning starts all over again.

However, tonight’s training session goes well and as it draws to a close, I’m observing the ‘07 (players born in 2007); the intensity is frantic as tackles fly in left, right, and centre. However, these periods of ferocity are punctuated as players break into tears. More often than not the pain isn’t physical, but mistakes bring gasping sobs and injuries are feigned to cover over the emotional turmoil. I turn to Matthias and ask if this is normal, he nods… It’s hard not to be impressed by the speed and skill of the play, but it’s equally hard not to despair that kids of 10 years old are feigning injury and crying because they’ve made a meaningless mistake… It’s obviously not meaningless to them;, again I’m left wondering why?

After we’ve packed up and said our good-bye to the kids, I’m standing with Ben and watching another session when Mike approaches. Mike is Nick’s Dad; Nick is one of the most skilful players in the ’07s and he’s also, often, bursting into tears. Mike is always dressed in fresh, fashionable athletic apparel, like the limited addition stuff sports brands make, his trainers always seem brand new and his hair is slicked back. I’ve been told he’s a CEO of some company and he’s infamous among the coaches for the time he threw his phone and wallet in apparent disbelief when Ben made substitutions which, in Mike’s eyes, weakened the team and jeopardised the result of a tournament game. He’s not unfriendly, however when I arrived at the club, he quizzed me, asking that I compare the 10-year olds at FC Barcelona with his son’s group. Today, he talks quietly as we discuss the competition in the team and the decision to increase the age of the academy.

> Mike: I think the atmosphere can be quite tough sometimes I hear, Nick tells me…. they are nice to each other, but they can all be tough to each other, because they are like Alphas, they are winners they wanna be tough guys and I’m the next Messi and sometimes that has an

impact on the atmosphere, but I think that's also life.

(Field note: Informal conversation, May 14th, 2017)

Comparison and internal competition are themes I'm familiar with, a conversation with a friend at the English Institute of Sport comes to mind and echoes this sentiment:

Matt: It's my biggest concern as a parent (in sport) and you can see it with Charlie… you can see it everywhere, as you say… the status anxiety or social judgments continually going on, you know kids are continually comparing themselves to each other, and yeah (sighs)… it just shuts so much down, it's so unhealthy but it's so hard to… you know… it's a classic again because you can't have that conversation with a 9 year old, so you've got to shape a different environment, you can't explain it rationally.

(Field note: Informal conversation, November 17th, 2017)

Talking to Mike by the side of the pitch I try to suggest that maybe it doesn't have to be the way it is?

James: I think that's what this new structure in the academy is about… Maybe it doesn't need to be like that at 10 years old… I mean you look at some of the best players… some of the most intelligent players, like the Xavi's, Iniesta's and Messi's they're not necessarily Alphas.

Mike: No, they are pretty low-key guys.

James: No one wants to lose, everyone wants to play the game, and be competitive and win… but it also doesn't matter enough that your coming off crying… because that's a learning opportunity and that's what I worry about, where is that pressure coming from?

Mike: Well, that's coming partly from the parents definitely…

Ben: And the culture around the academy…

Mike: I think there is a problem, like with us parents of course there's an expectation that the team should always win… of course there is… then of course… not always win, but I mean when you lose a game 5-0 to Nakdala all the parents are like, okay what the hell is happening? People say, "we're an academy, we have such great players how we can lose to Nackdala 5-0", when we know that we could beat them 8-0 the next day if we just have our head screwed on.

(Field note: Informal conversation, May 14th, 2017)

I recall the game in question and experience the hollow feeling accompanying the memory. I remember feeling “the mood is so serious and so toxic even I feel 'bad' standing on the sideline” (Field note, 29th April 2017). At half time, we had players in tears and during the second half we had to substitute one of our players as he aggressively, continuously and violently fouled (and hurt) opposition players. After leaving one opponent gasping for breath, our player refused to apologise saying the Swedish version of: ‘I don’t give a crap’ (Field note, 29th April 2017). At the end of the game our captain ripped the armband from his shoulder and winding up like a baseball pitcher threw it to the ground before storming off in tears. At the end of the game, players either sobbed on the ground or stormed around kicking, screaming, and swearing. On the train home I remember feeling shaken… I had to ring a friend to clear my head… “this isn’t a healthy environment for players or coaches”. I'd heard Pontus and Stephen talking about issues with performance anxiety… but to witness it in such a tangible way was horrible. It reminded me of what Johan Fallby said at the Stockholm Sports Federation’s Conference.

> Fallby said: I’ve been in junior teams’ dressing rooms that are more filled with more anxiety than the first team before the Champions League play-offs (at FC Copenhagen).
>
> (Field note: Headnote 29th April 2017)

Even away from academy duties these (controlling) behaviours seem to persist in some academy players. At a recent holiday program, three academy players oscillated between tearful tantrums and authoritarian outbursts.

> The captains of their respective teams and the oldest players at the camp, the behaviour of the academy players became more extreme and embarrassing as the morning wore on. They verbally abused the younger kids in their team before breaking down into tears if they lost a game. At one point a 7-year-old boy left the pitch in tears after an eleven-year-old academy player screamed at him after the final whistle – they were supposed to be teammates.
>
> (Field note: Headnote: 6th April 2018).

At 7 years old this kid was humiliated, shamed, and initiated into the world of football by an academy player he probably looked up to. I recall Stephen telling me the average age for kids to drop out of football has fallen to 9 years old in the north of Stockholm. I can see why.

4.5 Part 4 Summary

Part 4 begins with a coach meeting and illuminates the “English and Italian influences” on football in Sweden, alongside the tendency for coaches to coach the way they were coached. The significance of English football on and off the pitch is highlighted by Todd, while a formal conversation with Edvin introduces the lasting influence of AIK’s championship winning English coach Stuart Baxter. The Italian influence is observed with observations of smoke-filled stadiums delaying kick offs.

A focus on “Playing style” follows on from the previous section by highlighting the prevalence of a characteristically Italian defensive tactic employed throughout AIK, “shape”. In this section, tactics and game observations are interspersed with conversations about the Swedish mentality. The linear emphasis correlates with Stephens’ reflection on Swedish history and my own observations of the

“blinkered” play at a holiday camp. The end of this section highlights the current tension between two football paradigms. The 1970s British (almost Newtonian) paradigm is predicated on the belief that we can predict and control human behaviour and is abundant with machine metaphors. In contrast the evolution of Cruyff’s positional game is captured by Spanish coach Juanma Lillo.

“The illusion of control” introduces conversations that suggest, that many of AIKs core activities and organizational structures are predicated on the 1970s football paradigm. Discussions about AIKs first team are introduced alongside an interpretation of junior teams’ operational plans, which are surmised like this: *so much of this stuff is about punishing children.* Linear planning and administration are introduced as highly valued attributes within the Swedish Elite Football Federation, who grade football academies based on their administration, leading Stephen to exclaim *that is fucking Sweden, points for administration!*

The penultimate section “In the academy” highlights the academy coaches’ voices and opinions; in particular, a perception that: *When we have coaches’ meetings, we speak about organisation not football.* As this section concludes, informal conversations highlight how pressures trickle down leading to controlling hyper-organised coaching.

The final section “On the pitch” begins by highlighting the complex reality of coaching. Next an informal conversation highlights player perception of the environment: *I think the atmosphere can be quite tough sometimes I hear, Nick tells me…. they are nice to each other, but they can all be tough to each other.* The narrative concludes with observations of similar behaviours at a holiday camp… with some AIK academy players verbally abusing younger kids and breaking down into tears themselves when they lose. *At one point a 7-year-old boy left the pitch in tears after an eleven-year-old academy player screamed at him after the final whistle – they were supposed to be teammates.* The narrative finishes with the recognition that the average age that kids drop out of football has fallen to 9 years old. In football, like many other areas of Stockholm life, an emphasis on social comparison, status, judgment and competition seems to be suppressing the opportunity for open, potentially creative, collaboration.
